# Comparison of single bacteria and a bacterial reference community in a test against coated surfaces of varying copper content

**DOI:** 10.3389/fmicb.2025.1659828

**Published:** 2025-08-26

**Authors:** Yen Ly-Sauerbrey, Ronja Anton, Laura Kopruch, Carolin Luisa Krämer, Alessa L. Boschert, Claudio Neidhöfer, Oliver Schwengers, Daniela Zander, Stefan Leuko

**Affiliations:** ^1^Institute of Aerospace Medicine, German Aerospace Center, Cologne, Germany; ^2^Institute for Frontier Materials on Earth and in Space, German Aerospace Center, Cologne, Germany; ^3^Chair of Corrosion and Corrosion Protection, Foundry Institute, Division of Materials Science and Engineering, RWTH Aachen University, Aachen, Germany; ^4^Department of Natural Sciences, University of Applied Sciences Bonn-Rhein-Sieg, Rheinbach, Germany; ^5^MVZ Laboratory Dr. Limbach and Colleagues eGbR, Heidelberg, Germany; ^6^Institute for Medical Microbiology, Immunology and Hygiene, University Hospital of Cologne, Cologne, Germany; ^7^Division of Clinical Bacteriology and Mycology, University Hospital of Basel, Basel, Switzerland; ^8^Department of Bioinformatics and Systems Biology, Justus Liebig University Giessen, Giessen, Germany

**Keywords:** antimicrobial materials, copper coating, bacterial community, copper gradient, microbial load

## Abstract

**Introduction:**

Pathogens can easily transmit via surfaces and objects. In light of the ongoing pandemic of antimicrobial resistance, silently threatening millions worldwide, this is of particular concern in clinical and public environments. Thus, it is crucial to understand how antimicrobial materials influence surface-associated microbes and microbial communities. Copper, known for its antimicrobial activity, has demonstrated effectiveness against numerous clinically relevant pathogens. However, these *in vitro* pure cultures are in stark contrast to the *in vivo* microbial communities. Additionally, the application of pure copper surfaces is high in cost and maintenance.

**Methods:**

Hence, in this study we not only tested the antibacterial effectivity of different copper concentrations against single species, but also against a reference bacterial community representing the most abundant bacterial genera in public transport. This allowed a comparison of the antibacterial efficacy of copper against a bacterial community and against single species. Coatings on glass, which were composed of full copper (100 at.% Cu) and copper-aluminum alloys with different Cu contents (79 at.%, 53 at.% and 24 at.%) were tested with two selected single species (*Burkholderia lata* DSM 23089T and *Staphylococcus capitis* DSM 111179) and those species within the bacterial community.

**Results:**

In general, the survival of the two species within the bacterial community was higher compared to their respective survival as a single species, significantly for *S. capitis*. Surfaces with 100 at.% copper content showed the greatest antibacterial effect in terms of bacterial survival, with a reduced survival of up to 10^−6^. The 79 at.% Cu coating only had an inhibitory effect on the metabolic activity of *B. lata* when exposed to the surfaces as single species.

**Discussion:**

Our results highlight the benefits of additional testing of microbial communities rather than pure cultures. These experiments allow for enhanced evaluation of antimicrobial surfaces since they also take complex and diverse interactions within a surface microbiota into account. Therefore, community testing might be the more holistic approach for the testing of antibacterial materials.

## 1 Introduction

Microbial dispersion naturally accompanies human presence. Through breathing, bioaerosols are spread, and direct contact to the environment causes the distribution of microbes that are harboring the skin. While most microorganisms are harmless or even beneficial, caution is needed when it comes to the spread of (opportunistic) pathogens. The currently ongoing silent pandemic of antimicrobial resistance (AMR) which includes multidrug-resistant bacteria (MDR), is already affecting millions of patients, resulting in limited or no treatment option of infections (Mahoney et al., 2021; Naghavi et al., 2024). Public surfaces play a vital role in the spread of pathogens (Xiao et al., 2019). Therefore, countermeasures to reduce infection transmission are needed. Transmission of pathogens and microorganisms in general is facilitated by many factors. Especially crowded environments, but also confined spaces lacking proper ventilation are hotspots of antimicrobial spread. Public transportation in large cities with millions of daily commuters combines both of the aforementioned features. Hence, it is a key environment for microbial transmission, simultaneously also highlighting the need for effective countermeasures.

Different countermeasures are available to reduce the microbial spread. In terms of air cleaning, disinfection can be performed using fumigation of e.g., hydrogen peroxide (Møretrø et al., 2019). For material disinfection, UV-disinfection, is common in hospital settings (Guettari et al., 2021; Ghosh et al., 2022). Antimicrobial materials are another approach to reduce the bacterial load on fomites. A great variation of antimicrobial materials and surfaces are available and have different antimicrobial mechanisms. For instance, some anti-biofouling surfaces reduce the attachment of microorganisms to the surfaces (Linklater et al., 2021), while others have biocidal species that inactivate microorganisms, like biocidal nanocomposites (Tamayo et al., 2016). Besides biocidal reactives, some surfaces rupture bacterial cells (Luan et al., 2018). Other commonly used antimicrobial materials are metals. So-called “metalloantibiotics” can cause toxicity for cells (Frei et al., 2023). However, while some metals exhibit antimicrobial properties, certain bacteria possess or have developed resistance to high metal ion concentrations (Hobman and Crossman, 2015). Therefore, deliberate and strategic application of these materials is essential.

One of the best-known antimicrobial metals is copper. Although copper is essential for the cells of all aerobic organisms (Festa and Thiele, 2011), toxic concentrations damage cells in various ways. Hence, while copper ultimately results in cell inactivation or death, the point of attack can vary between different types of microorganisms. For instance, viruses are affected by RNA degradation and membrane damage (in enveloped viruses) caused by copper ions. In fungi, on the other hand, copper ions cause physical damage of the cell membranes (Salah et al., 2021). In bacteria, copper not only damages the cell membrane, but also the DNA, due to the intrusion of copper ions and resulting oxidative stress (Grass et al., 2011; Santo et al., 2011; Pramanik et al., 2012). Oxidative stress involves reactive oxygen species (ROS), which are generated by both Fenton-like and Fenton-independent reactions, which cause oxidative damage of the cells through lipid peroxidation, protein oxidation, the above mentioned DNA and membrane damage, ultimately leading to cell death (Barnham et al., 2004; Pham et al., 2013; Salah et al., 2021).

Since copper functions as a cofactor, which catalyzes many redox reactions, high concentrations pose an issue with the copper homeostasis within the cells. High concentrations of copper can become toxic, since it can replace the iron in iron-sulfur clusters of proteins and damage or inactivate those proteins (Chillappagari et al., 2010).

Interestingly, the mechanism behind the antimicrobial copper surfaces is different between wet- and dry-contact killing (Ramos-Zúñiga et al., 2023). While it is not yet fully understood how copper inactivates cells in a wet contact killing setting, even less is known about the mechanism behind dry contact killing (Grass et al., 2011). However, it has been observed, that cells accumulate copper ions much faster during dry contact killing compared to wet contact killing, leading to damage especially of the cell membrane (Santo et al., 2011).

The application of copper as an antimicrobial element dates back to ancient times and has been studied extensively. Nowadays, it is used or tested in different settings, such as in hospitals (Mikolay et al., 2010) or in public transport (Williams et al., 2024). Nevertheless, copper is not a cost-effective element for processing and long-term use. It corrodes easily, and it is therefore not favored to be applied in its pure state. A promising method of enhancing the durability while maintaining cost efficiency is the use of copper alloys. In a past study, the effective antimicrobial copper content was tested by using materials with different copper contents in a hospital setting (Karpanen et al., 2012). In the described study by Karpanen et al. (2012), copper alloys with ≥ 58% copper content resulted in a microbial reduction on surfaces. Another study by Mehtar et al. (2008) tested different surfaces against nosocomial pathogens and a minimum copper content of > 55% was determined.

In this study, glass slides were used since glass is not antimicrobial and therefore does not influence the effect of the coatings. Further, the used glass substrates support reproducibility due to their standardized roughness, which could otherwise influence the settling and behavior of the tested bacteria. Nevertheless, this approach can be applied to various materials. Another benefit of the used coatings is that aluminum is used in the alloy, which is more cost effective than zinc in brass (Westmetall, 2024)^1^ which is also the subject of investigations into antimicrobial materials (Timofeev et al., 2025). Further, pure copper oxidizes quickly, which creates a colored surface making the material less user-friendly. Hence, copper-aluminum (Cu-Al) alloy coatings are considered to be highly suitable for real-setting applications.

To test the effectivity of such materials, many studies use microbial model organisms. A common issue of many studies is not the microbial selection for testing, but rather the applicability to real-world settings. A natural microbial environment is shaped by a variation of microorganisms, and the species within a microbial community interact. Microbial communities are complex, therefore studying the effects and the interactions within a microbial community is challenging (Pierce and Dutton, 2022). Many studies exist, that investigate the effect of heavy metals, such as copper, on soil microbial communities (Corcoll et al., 2019; Sutcliffe et al., 2019; Shaw et al., 2020), but the investigation of the surface microbiome of highly touched surfaces is still limited. Yet, especially in hospital settings with an already high selection pressure due to antibiotics and regular disinfection, the risk of giving multi-drug resistance bacteria a further advantage by reducing the total bacterial load of surfaces, has to be considered.

During this study, not only single species, but also a defined bacterial community which simulates the microbiome of public transport was used. This particular bacterial community (Ly et al., 2024) has already been successfully applied as a reference for other experimental settings (Kohl et al., 2024). This approach allows the investigation of differences between single species and a bacterial community after the exposure to copper and copper alloy surface coatings with different copper contents (24 at.%, 53 at.%, 79 at.%, 100 at.%). It enhances our understanding of the antimicrobial effect of copper and deepens the knowledge on what parameters may affect such experimental setups.

The findings of this study aim to contribute to research focused on reducing microbial spread while simultaneously minimizing material costs (full copper vs. copper alloy) and the economic impact of multidrug-resistant (MDR) bacteria.

## 2 Materials and methods

### 2.1 Used organisms and preparation of bacterial suspensions

The human skin associated bacterium *S. capitis* was selected for this study, as it is one of the most common genera found in indoor environments. The used strain of *S. capitis* DSM 111179 (*S. capitis*) harbors an antibiotic resistance against rifampicin, which allows selective determination of survival within the simulated bacterial community. Another tested bacterial strain was *B. lata* DSM 23089*^T^*, as a biosafety level 1 strain of *Burkholderia*, allowing straightforward handling. The genus *Burkholderia* is also commonly found in public transport environments. One characteristic of *B. lata* DSM 23089*^T^* (*B. lata*) is its resistance against streptomycin and therefore allowing selective growth. Both strains of *S. capitis* and *B. lata* were purposely chosen for testing, since their morphological composition and therefore the expected reaction to copper surfaces differed. *S. capitis* is gram-positive and coccoid, while *B. lata* is gram-negative and has rod-shaped cells.

The species and growth conditions of the bacterial community are listed in Table 1 and are described in greater detail in Ly et al. (2024).

**TABLE 1:** List of strains within the standardized bacterial community, growth conditions and references.

Strain	Growth conditions	References
*Burkholderia lata* DSM 23089^T^	R2A 37°C	Vanlaere et al., 2009
*Corynebacterium halotolerans* DSM 44683^T^	TSB/TSA 37°C	Chen et al., 2004
*Enterococcus viikkiensis* DSM 24043^T^	BHI 37°C	Rahkila et al., 2011
*Flavobacterium frigoris* DSM 15719^T^	R2A 20°C	Van Trappen et al., 2004
*Propionibacterium cyclohexanicum* DSM 16859^T^	BHI 37°C	Kusano et al., 1997
*Pseudomonas antarctica* DSM 15318^T^	TSB/TSA 20°C	Reddy et al., 2004
*Sphingomonas rubra* DSM 26135^T^	R2A 20°C	Huo et al., 2011
*Staphylococcus capitis* DSM 111179	R2A 37°C	Sobisch et al., 2019
*Streptococcus halotolerans* DSM 101996^T^	BHI 37°C	Niu et al., 2016

R2A, Reasoner’s 2A; TSB, tryptic soy broth; TSA, tryptic soy agar; BHI, brain heart infusion.

Bacterial strains were grown for 18 h at the respective growth conditions listed in Table 1. The bacterial cells were then harvested in the stationary growth phase. Afterward, the bacterial cultures were centrifuged at 323 *g* for 10 min, and the supernatant was discarded. The cell pellet was resuspended in 50 mL in phosphate buffered saline (PBS; Na_2_HPO_4_ 7.0 g, KH_2_PO_4_ 3.0 g, NaCl 4.0 g, per Liter, pH 7.5). The washing step was repeated once. The cell solutions were set to a concentration of 10^7^ cells/60 μL after OD_600__*nm*_ measurement. These steps were followed by colony forming unit (CFU) determination on the respective media given in Table 1 to confirm the cell concentration. For the bacterial community, all nine bacterial strains were mixed in equal cell counts with a final concentration of 10^7^ cells/mL.

### 2.2 Copper and aluminum coatings on glass surfaces

The coatings were applied to microscopy slides as substrate material (Labsolute, Th. Geyer) with sample dimensions of 76: 76: 1 mm. Batch magnetron sputtering (Z400, Systec SVS vacuum coatings, Karlstadt, Germany) was applied for deposition of the coatings. Prior to deposition, one side of the glass substrate was subjected to Ar^+^ ion etching for sample surface cleaning and activation. The target material used were dense polycrystalline targets of Cu and Al with a diameter of 100 mm. In order to obtain coatings with different Cu contents, DC co-sputtering was performed at different target powers. The total pressure during deposition was 0.4 Pa in an Ar atmosphere with a constant flow rate of 20 sccm. The coating thickness was 0.8–2.0 μm.

The sputtered coatings were analyzed using an X-ray diffraction (Bruker D8 Advance, Cu Kα radiation, EVA/Topas 4.2 software package, Bruker AXS, Karlsruhe, Germany), which revealed the phases within the deposited coatings. In order to determine the coating thickness, cross sections were analyzed using a scanning electron microscope (SEM) (DSM Ultra 55, Carl Zeiss NTS, Wetzlar, Germany). Additionally, energy-dispersive X-ray spectroscopy (EDS) (Aztec, Oxford Instruments, Abingdon, United Kingdom) was executed at 5 kV to determine the coating content of Cu and Al.

### 2.3 Wet contact killing

**FIGURE 1:**
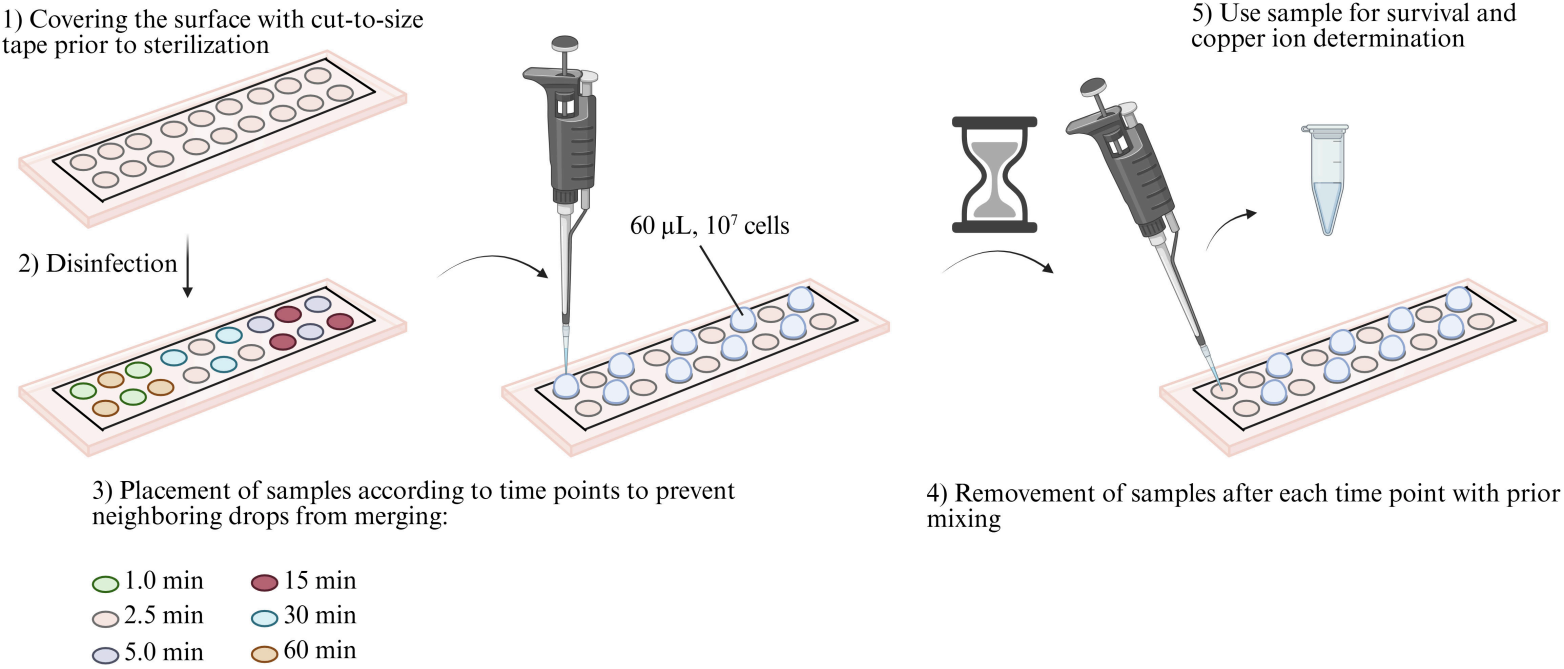
Experimental procedure of wet contact killing assay. (1) First, the surfaces were covered with cut-to-size tape with 18 cut-outs with a diameter of 6 mm each. (2) The surface was then disinfected with 99% ethanol and irradiated with germicidal UV-C (254 nm) for 30 min. (3) Then, 60 μL (10^7^ cells) of the prepared bacterial solution was applied with a certain time delay to ensure efficient work without the samples merging due to insufficient distance between them. (4) After each time point, the samples were mixed before being transferred to tubes. (5) The obtained samples were then used to determine the survival rate, perform the metabolic activity assay, and ICP-MS measurements.

**FIGURE 2:**
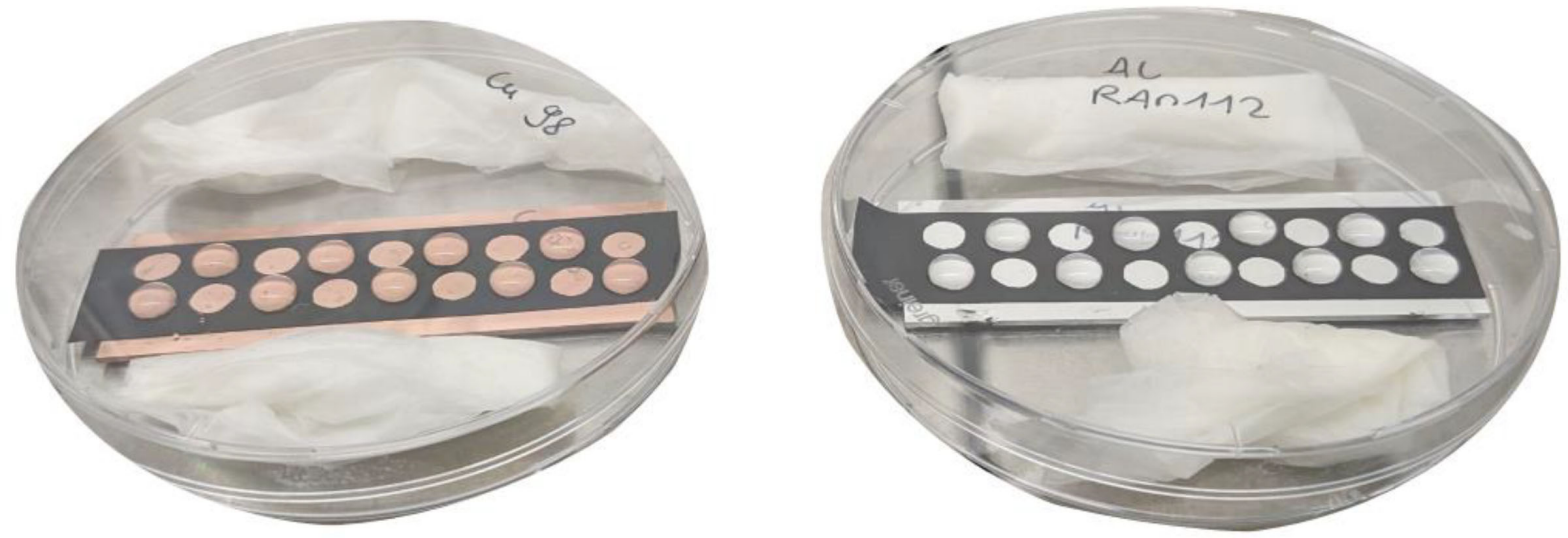
Experimental setup during wet contact killing, exemplary on a copper (100 at.%) and aluminum (100 at.%) surface. During the experiment (after step 3 of Figure 1), the surfaces were positioned in petri dishes packed with wet paper towels to maintain constant high humidity. This reduced evaporation of the samples.

An overview of the experimental procedure is displayed in Figure 1. To prepare the surfaces for the wet contact killing assay, adhesive PVC tape was cut out to defined spots of 6 mm diameter for each bacterial sample, and the surfaces were disinfected with 99% ethanol and subsequent UV-C (254 nm) treatment for 30 min. The microbial samples were prepared in PBS, and 60 μL (10^7^ cells/60 μL) were applied on each surface spot for 1, 2.5, and 5 min for *B. lata*, and 15, 30, and 60 min for *S. capitis*. Time points were selected based on survival results of preliminary experiments and allowed the detection of survival within the tested time scale. Of the retrieved samples, 10 μL were used for the determination of bacterial survival, 10 μL for metabolic activity assay, and 10 μL for ICP-MS analysis. The wet contact killing assay was conducted using a minimum of three biological replicates. The experimental setup during the experiment is displayed in Figure 2.

### 2.4 Determination of bacterial survival

To determine the bacterial survival, a 10-fold serial dilution of each sample was prepared from 10^–1^ to 10^–8^ in PBS and 20 μL of each dilution was plated on respective media plates. For single species testing, R2A agar plates were used. For samples of the bacterial community, selective growth of *B. lata* was performed on R2A containing streptomycin (150 μg/mL), and for *S. capitis* R2A with rifampicin (5 μg/mL) was used. Agar plates were incubated at 37°C for 48 h. Colony forming units (CFU) were counted afterward to determine the CFU/mL. The survival fraction N/N_0_ was then calculated by dividing the CFU/mL of the treatment conditions by the CFU/mL of the initial cell solution.

### 2.5 Copper ion content detection via ICP-MS

One method of analysis after wet contact killing experiment was the determination of the Cu 63 isotope with Inductively Coupled Plasma - Mass Spectrometry (ICP-MS) analysis, using a NexION^®^ 2000 ICP-MS (Perkin Elmer, Massachusetts, United States). Measurements were performed in the kinetic energy discrimination (KED) mode using Ar (99.999 vol%) as the nebulizer gas and He (99.9999 vol%) as the collision gas. The cell solution was diluted by a factor of 600, using 10 μL of the solution and diluting it in 5,990 μL of 0.69% HNO_3_. PBS was used as control sample. For ICP-MS, triplicate samples were used (*n* = 3).

### 2.6 Determination of metabolic activity via resazurin reduction

To investigate the metabolic activity of the bacterial cells after exposure to the tested surface materials, the reduction of resazurin was used as indicator (alamarBlue^®^ Cell Viability Reagent, Thermo Fisher™). This method was used already by Cortesão et al. (2021) with fungal spores. The assay was conducted in a 96-well plate, with a total volume of 200 μL reaction mix per well. The reaction mix consisted of 170 μL culture media with and without antibiotics, as described in see section “2.1 Used organisms and preparation of bacterial suspensions,” 10 μL bacterial solution, and 20 μL of alamarBlue. The 96-well plate was incubated at 37°C for 24 h. The OD_600nm_ and OD_570nm_ were measured every 30 min using a Multi-Detection Microplate Reader (Infinite M200 PRO, Tecan). Before each measurement, the samples were subjected to orbital shaking. To calculate the percentage of resazurin reduction, the standard protocol of the manufacturer was used (Thermo Fisher Scientific, United States)^2^. Each reaction was conducted in biological triplicates (*n* = 3).

### 2.7 Scanning electron microscopy (SEM)

For the phenotypical analysis of the cells after wet contact killing, scanning electron microscopy was conducted. Sample preparation included the wet contact killing procedure with coatings of 100 at.% Cu and Al, as described in see section “2.3 Wet contact killing,” except for the recovery of the samples. The cell solutions were dried on the surfaces for 2 h, followed by fixation using 2% paraformaldehyde (PFA; Merck 104005) in 0.05 M HEPES buffer (pH 7.2) for 1 h (30 min at 25°C + 30 min at 37°C). One exception was made for the Cu surface with the bacterial community. Here, the contact time was 1 h and after fixation, the surface was washed three times with PBS and one time with sterile, deionized water.

To examine the surfaces after wet contact killing, a scanning electron microscope (SEM) (DSM Ultra 55, Carl Zeiss NTS, Wetzlar, Germany) was used with the SE detector with a voltage of 5 kV. Prior to the investigations the surfaces were Pt-coated for 80 s to create a conductive surface.

### 2.8 Sequencing of *B. lata* DSM 23089^T^

To identify genes which are potentially related to copper defense or tolerance within the genomes of *B. lata* and *S. capitis*, the following process was conducted. The genome of *S. capitis* DSM 111179 was already submitted by the German Aerospace Center and is accessible as NCBI RefSeq assembly GCF_025272695.1 on NCBI. For *B. lata* DSM 23089^T^, no whole genome sequence data was available. We conducted nanopore sequencing as well as whole genome sequencing prior to hybrid assembly. For whole genome sequencing, DNA was extracted using the QIAGEN Genomic-tip 20/G in combination with the Genomic DNA buffer Set, according to the manufacturer’s instructions (QIAGEN, Hilden, Germany). Further processing for Illumina sequencing was performed by the Cologne Center for Genomics as described in the following. Libraries were prepared with the TruSeq DNA Nano library preparation kit (Illumina)^3^ without PCR, starting with 1 μg gDNA input. Library preparation was followed by clean up and size selection using SPRI beads (Beckman Coulter Genomics) aiming for an insert size of 350 bp. After library quantification (Qubit, Life Technologies) and validation (Tape Station, Agilent), equimolar amounts of library were pooled. The library pool was quantified by real-time PCR using the Peqlab KAPA Library Quantification Kit and the Applied Biosystems 7900HT Sequence Detection System and then sequenced on an Illumina NovaSeq6000 sequencing instrument with a paired-end 2 × 150 bp.

Nanopore sequencing was conducted by Noscendo GmbH, Germany. DNA isolation, library preparation, and sequencing on the MinION benchtop sequencer were carried out as described in Neidhöfer et al. (2023). All genomes were assembled following a hybrid long-read-first assembly approach using Hybracter v0.11.0 (Bouras et al., 2024). Resulting genomes were annotated using Bakta v1.11.0 (Schwengers et al., 2021) via Bakta Web (Beyvers et al., 2025).

### 2.9 Statistical analysis

Student’s *t*-tests were performed to calculate the level of significance between treated and untreated samples. Untreated samples were not exposed to any wet contact killing and treated samples were exposed to wet contact killing. If the equal variance test (Brown-Forsythe) failed between the two data sets, Welch’s *t*-test were performed instead of Student’s *t*-test, since the latter assumes that the variances of the two samples are equal. If the data of both data sets were not normally distributed, the Mann-Whitney Rank Sum test was performed instead, as this test makes no assumptions about the distribution of the data. To calculate the significant differences within the alamarBlue data sets, two-way Repeated Measures (RM) ANOVA was performed to compare the positive control to respective bacterial samples. The control samples (PBS) of the ICP-MS measurements were compared to the bacterial samples by paired *t*-test. Significant differences were defined with *p* ≤ 0.05 (*), *p* ≤ 0.01 (**) and *p* ≤ 0.001 (***). All statistical analyses were executed with SigmaPlot 14.5 (Systat Software Inc).

## 3 Results

### 3.1 Analysis of coating

The deposited coatings were crystallin after deposition. The phases of the coatings formed during processing were determined using X-Ray measurements. The 100 at.% Cu coating and the 100 at.% Al coating consisted of only one phase of Cu or Al. For the alloy coatings with variation of Cu content intermetallic phases of Cu and Al were analyzed.

In order to determine the thickness of each coating type, cross sections were investigated in the SEM. Table 2 shows the coating thicknesses. Due to its ductility, Cu is difficult to prepare by metallographic preparation methods, therefore the coatings had a slight difference in thickness as a sputtering rate could not be determined without intensive preparation methods. However, this subtle variation in thickness was not expected to play any role in the antimicrobial behavior.

**TABLE 2:** Coating thickness of each coating type measured in cross section via scanning electron microscope (SEM).

Coating (Cu content in at.%)	Coating thickness (μm)
100	∼1.4
79	∼0.8
53	∼1.4
24	∼0.8
0	∼2.0

### 3.2 Bacterial survival on surfaces with different copper contents

**FIGURE 3:**
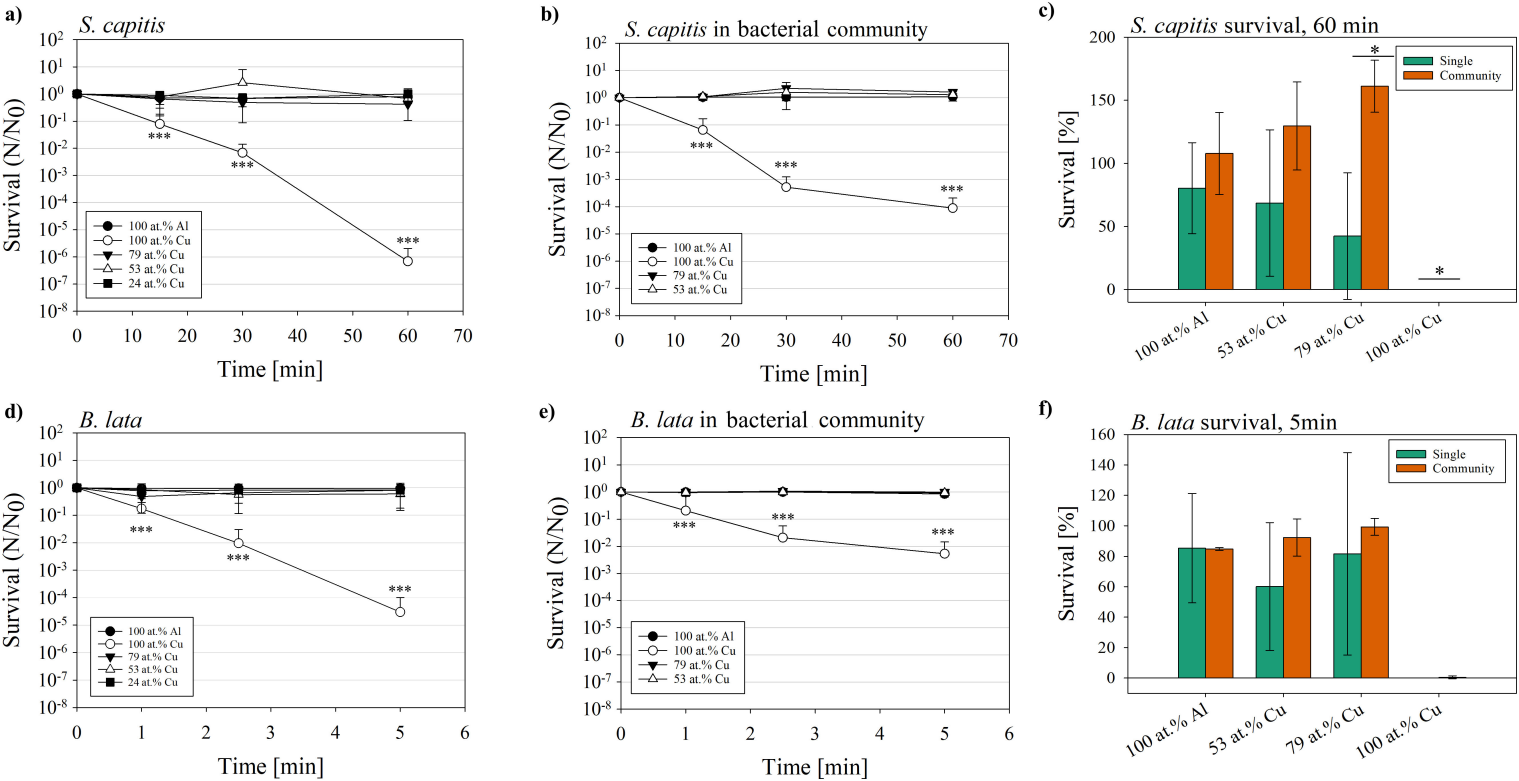
Survival fraction (N/N_0_) of the species *S. capitis* and *B. lata* after exposure to glass slides coated with copper gradients of 100 at.%; 79 at.%, 53 at.%, and 24 at.%, or coated with 100 at.% aluminum as negative control. Survival fraction of *S. capitis* as **(a)** single species and **(b)** within the bacterial community, survival **(c)** after 60 min of contact killing. *B. lata* as **(d)** single species and **(e)** within the bacterial community, and **(f)** survival after 5 min of contact killing. Results shown are mean and standard deviation of three biological replicates. Statistically significant differences in survival fraction relative to the 100 at.% Al control **(a,b,d,e)**, and the differences in survival between single species and respective species within the bacterial community **(c,f)** are displayed. Significant differences were defined with *p* ≤ 0.05 (*), *p* ≤ 0.01 (**) and *p* ≤ 0.001 (***).

The bacterial community and the single species *S. capitis* and *B. lata* were exposed to a variation of surfaces for different time periods and their survival was determined. Results of the survival, survival fraction N/N_0_ and significance tests are summarized in Supplementary Tables 1–7. Over the maximum time tested of 60 min, the survival fraction of *S. capitis* was significantly reduced (*p* < 0.001) on the 100 at.% copper coating in both tested scenarios (single vs. within bacterial community, Figures 3a, b). Comparing the survival of *S. capitis* as single species and within the bacterial community, first differences were observed from the second time point at 30 min. Here, the survival reduction as single species was 2-log scales (10^–2^), while within the bacterial community, the survival fraction dropped to less than 10^–3^ cells/mL. Contrarily, after 60 min of exposure, *S. capitis* showed less reduction within the bacterial community when compared to its testing in pure culture. This observation was reflected in a difference of 2-log scales. The direct comparison of single species and bacterial community survival on the different surface types was conducted for the last time point (60 min) (Figure 3c). On the one hand, no significant difference was observed between *S. capitis* as single species and within the bacterial community after contact killing on 100 at.% Al and 53 at.% Cu. On the other hand, a significant difference was observed for the 79 at.% Cu (*p* = 0.021, survival single 42.3%; within bacterial community 161%) and 100 at.% Cu surface (*p* = 0.013, survival single 6.9 × 10^–5^%; within bacterial community 8.8 × 10^–3^%). Therefore, *S. capitis* showed improved survival within the bacterial community.

The survival fraction of *B. lata* was determined based on the exposure of the bacteria for 1, 2.5, and 5 min (see Figures 3d, e), based on preliminary experiments conducted, which showed no detectable survival of *B. lata* after 15 min of 100 at.% Cu surface contact (data not shown). Within (Figure 3d), the survival fraction is displayed for the testing of *B. lata* as a single species. Several similar trends were observed in the survival of *B. lata* as a single species as well as within the bacterial community when compared to *S. capitis*. For one, they were both significantly affected in the survival by the 100 at.% Cu coatings (*p* < 0.001, survival fraction *B. lata* single 3.74 × 10^–5^). This was also the case for *B. lata* within the bacterial community (*p* < 0.001, survival fraction 5.33 × 10^–3^, Figure 3e). Focusing on the last time point (5 min), a 2.5-log discrepancy was shown between survival as single species and within the bacterial community. Another akin tendency between *S. capitis* and *B. lata* was that single strains and the strains within the community were affected similarly at the first time point after 1.0 min. The last time point (60 and 5 min) showed better survival of the bacteria within the community, also for both selected species (Figures 3b, e). However, as displayed in Figure 3f, there was no significant difference in survival after 5 min of contact killing between *B. lata* as singles species and *B. lata* within the bacterial community.

Overall, *B. lata* showed a higher susceptibility to the copper coated surfaces compared to *S. capitis*. Our results showed that both *B. lata* and *S. capitis* survived a longer exposure to copper surfaces better within a bacterial community compared to exposure as a single species. However, we only saw significant differences for *S. capitis*.

### 3.3 Metabolic activity after wet contact killing

To evaluate the metabolic activity of *S. capitis* and *B. lata* after exposure to the coated surfaces as single species and within the bacterial community, an alamarBlue assay was performed over 20 h.

**FIGURE 4:**
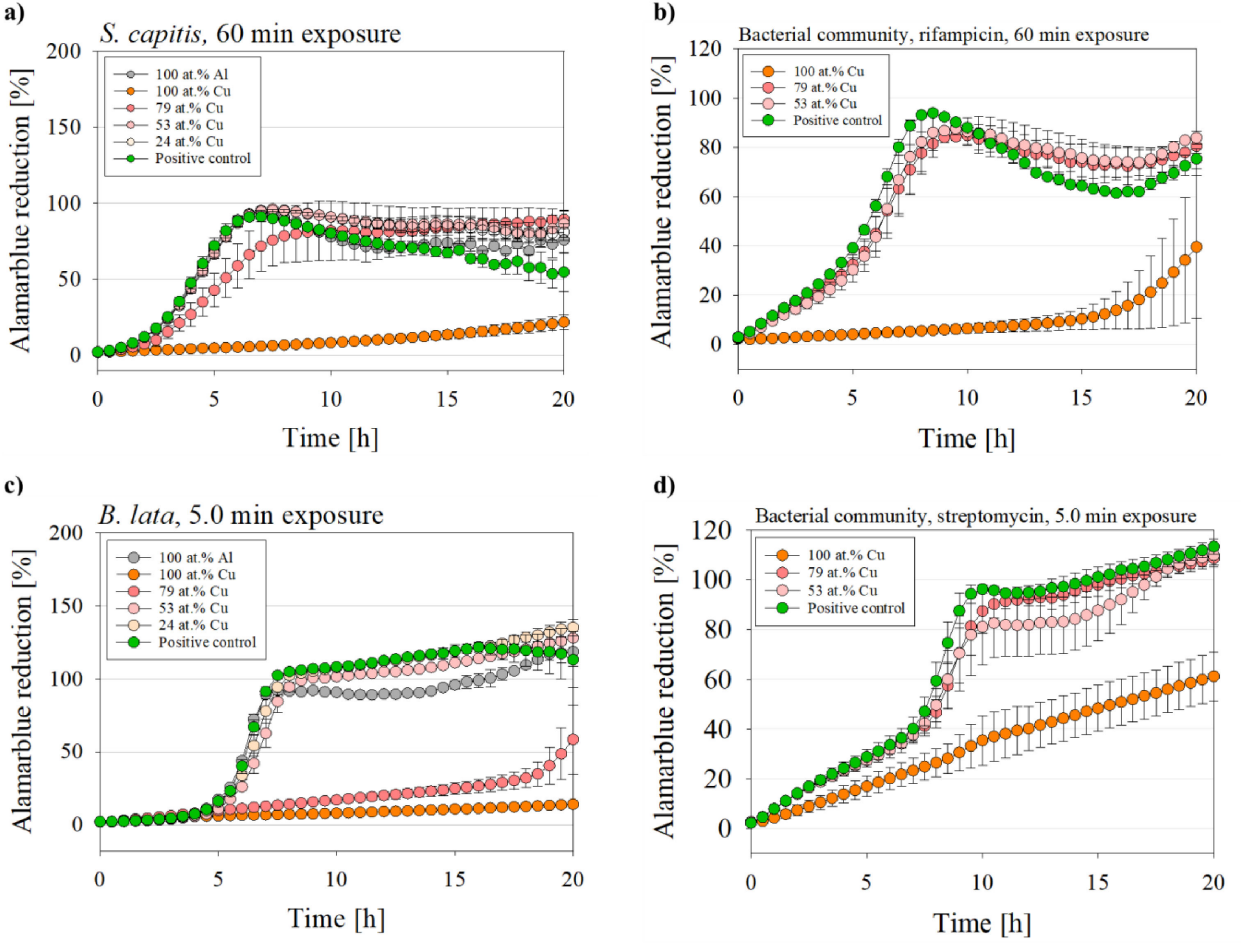
Metabolic activity of *B. lata* and *S. capitis* as single species and within the bacterial community after wet contact killing assay. The species **(a)** *S. capitis* and **(b)** *S. capitis* within the bacterial community (medium supplemented with rifampicin) were treated on the surfaces for 60 min. The species **(c)** *B. lata* and **(d)** *B. lata* within the bacterial community (medium supplemented with streptomycin) were tested for 5 min to the surfaces prior to the metabolic activity measurement. The contact killing was performed with coatings with varying copper content, aluminum and positive control (= untreated bacterial sample with no wet contact killing). The data is displayed as mean and standard error (*n* = 3).

Figure 4 displays the measured metabolic activity via alamarBlue assay of the single species *S. capitis* (Figure 4a) and *B. lata* (Figure 4c) and the species within the bacterial community after exposure to the surfaces and settings as described earlier. Significance was tested and p-values were summarized in Supplementary Tables 8, 9. In the alamarBlue assay for samples of the bacterial community, rifampicin was supplemented in the media in samples which were exposed to 60 min of wet contact killing for selective growth and measurement of *S. capitis* (Figure 4b). Streptomycin was added into the media of samples that were prolonged 5 min of wet contact killing to select for *B. lata* (Figure 4d).

Pure cultures of *S. capitis* (Figure 4a) that were exposed to either the control material 100 at.% Al, 53 at.% Cu, 24 at.% Cu, as well as the positive control (untreated bacteria) increased their metabolic activity exponentially during the early phase: The alamarBlue reduction for those samples reached nearly 100% after ∼7 h into measurement. Interestingly, the *S. capitis* single species 79 at.% Cu sample showed a delayed metabolic activity (8.5 h until maximum alamarBlue reduction). The greatest affected samples were those after exposure to the 100 at.% Cu coated surface, which, for the *S. capitis* single species only showed slight increase up to ∼22% after 20 h of incubation and displayed very significant (*p* = 0.003) differences compared to the positive control.

In contrast, *S. capitis* within bacterial community (Figure 4b) exposed to 79 at.% Cu and 53 at.% Cu reached a maximum alamarBlue reduction of 84.8% and 87.6%, respectively, only after 9–10 h of measurement. The positive control showed an even stronger alamarBlue reduction after 8 h. As for the 100 at.% Cu samples, the reduction started after approximately 15 h of incubation.

Figure 4c displays the metabolic activity of *B. lata* after 5 min of wet contact killing on all tested surface types, while (Figure 4d) shows it for *B. lata* within the bacterial community on 100 at.% Cu, 79 at.% Cu, and 53 at.% Cu. A maximum alamarBlue reduction was reached by the *B. lata* single species samples after ∼8 h into measurement for the positive control, 53 at.% Cu, 24 at.% Cu, and 100 at.% Al treated samples. For *B. lata*, pure cultures that were exposed to 79 at.% Cu surface, a linear increase of the metabolic activity was followed by an exponential rise after 18 h of incubation, resulting in a highly significant (*p* < 0.001; 20 h into alamarBlue: 79 at.% Cu 58%; PC 113%) difference compared to the positive control. Minimal increase of the alamarBlue reduction was observed for the 100 at.% Cu coated samples, resulting in a reduction of approximately 14% after 20 h of incubation and a very significant difference (*p* = 0.002; 20 h into alamarBlue: 100 at.% Cu 14%; PC 113%) compared to the positive control. Similar to the positive control, other samples, such as 53 at.% Cu and 24 at.% Cu, showed an alamarBlue reduction of over 100%. This was also observed in the advanced measurement times in Figure 4d. This oversaturation can be traced back to different reasons. For instance, accumulation of the reduced product resorufin, or the over-reduction of alamarBlue to hydroresorufin, which can be avoided by a shorter incubation time (O’Brien et al., 2000).

For the samples of *B. lata* within the bacterial community, the maximum metabolic activity was reached two hours later compared to the pure culture, after ∼10–11 h, for the positive control and 53 at.% Cu surface samples. A great difference between single species and bacterial community samples was displayed for the 79 at.% Cu and 100 at.% Cu surfaces. In contrast to the single species exposed to 100 at.% Cu with 14% maximum alamarBlue reduction, *B. lata* within the bacterial community resulted in a linear increase of alamarBlue reduction, with a final reduction of approximately 60% after 20 h of incubation. Further, the results of the 79 at.% Cu sample of the bacterial community were comparable to the 53 at.% Cu sample with increased metabolic activity, whereas as pure culture, the sample exposed to 79 at.% Cu showed significantly reduced metabolic activity.

To rule out any effects of the addition of the antibiotics, the metabolic activity assay was conducted without antibiotics too. The results showed no significant difference between the trend of the alamarBlue reduction (Supplementary Figure 3). However, other bacteria within the bacterial community could have contributed to the alamarBlue reduction in this case.

The metabolic activity of both *S. capitis* and *B. lata* reduced with increasing copper content, as indicated by decreased reduction of resazurin. The metabolic activity was also measured for each time point that was tested in section 3.1 (1 min, 2.5 min for *B. lata* and 15 min, 30 min for *S. capitis*), showing mostly similar behavior of the resazurin reduction (Supplementary Figures 1, 2).

To conclude this section, the metabolic activity assay showed that the 100 at.% Cu surface treatment led to a decreased alamarBlue reduction for all single species and bacterial community when compared to the positive controls. As for *S. capitis* single species, the maximum metabolic activity was 22% after 20 h into the assay. The samples exposed to the other materials showed high resazurin reduction after only 7.0–8.5 h. These results were similar for *S. capitis* within the bacterial community.

For the single species *B. lata*, a maximum alamarBlue reduction of 14% was reached after 20 h of measurement after wet contact killing on 100 at.% Cu. The sample treated with 79 at.% Cu surface also exhibited a significantly reduced metabolic activity with a maximum of 58% after 20 h of measurement. The other materials and the positive control showed peak reduction only after 10-11 h of measurement. Compared to the single species, the bacterial community samples showed higher metabolic activity for the 100 at.% Cu and 79 at.% Cu samples.

Lastly, the bacterial community samples (5 min and 60 min wet contact killing) treated with 100 at.% Cu surface coating, showed a maximum alamarBlue reduction of 40% and 60%, respectively. In contrast, the single species *S. capitis* and *B. lata* exhibited a maximum alamarBlue reduction of only 22% and 14%, respectively.

### 3.4 SEM imaging of bacterial cells on copper and aluminum containing coatings

**FIGURE 5:**
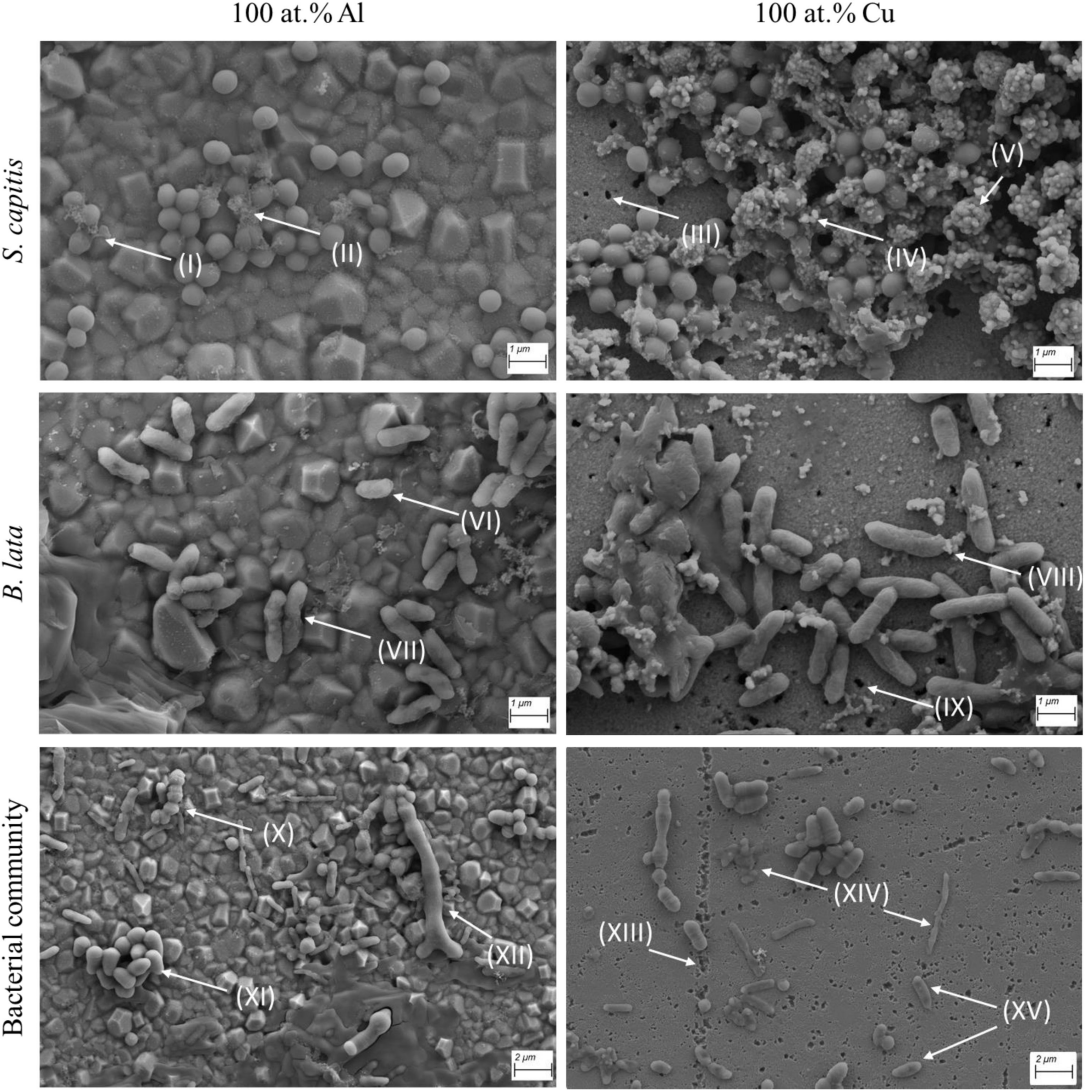
Scanning electron microscopy (SEM) imaging of *S. capitis, B. lata*, and the bacterial community on 100 at.% Al and 100 at.% Cu surfaces after 2 h of contact (1 h for the bacterial community on copper). (I, II, IV, V) cell debris, (III, IX, XIII) indication of surface degradation, (VI, VII, VIII, XIV) visibly damaged cells, (X, XI, XII, XV) intact cells.

While the results above present an overview of what effect the copper containing surfaces have on the survival and metabolic activity of the cells, SEM imaging of selected samples was conducted to gain further insights. Cell suspensions of *S. capitis, B. lata* and the bacterial community were applied on 100 at.% Cu and 100 at.% Al surface coatings, respectively (Figure 5). A contact time of approximately 2 h was selected for full evaporation of the sample prior to sample fixation for SEM. Only for the bacterial community sample on 100 at.% Cu, the contact time was limited to 1 h due to protocol optimization. This difference in time has to be taken into account for the following observations and consecutive comparison of the samples.

The exposure of *S. capitis* on surfaces containing aluminum and copper showed distinct differences: First, only little damage of the cells or products of the cells were observed for *S. capitis* on 100 at.% Al (I, II). In contrast, on 100 at.% Cu surface, cells of *S. capitis* were found surrounded or completely covered by potential cellular debris or other organic material (IV, V). However, the cells showed no striking anomalies when compared to the cells that were exposed to 100 at.% Al surface coatings. It was noticeable that degradation of the copper coatings occurred, leaving holes of varying diameters within the coatings (III).

For *B. lata*, which was observed to be the more susceptible species according to the survival after exposure on copper surfaces, morphological damage was visible for both surface types, aluminum (VI, VII) and copper (VIII). Interestingly, most cells seemed to be phenotypically intact. Similar to *S. capitis*, some extracellular debris was observed next to or on the cells on the copper coating. Additionally, signs of degradation in the surface coating were also observed (IX).

The last tested condition was the bacterial community. The different cell types and morphologies were observed (X, XI, XII), showing no morphological impact or damage on 100 at.% Al surface coating. Again, as with the two tested pure cultures, the surface of the 100 at.% Cu coating showed characteristics of degradation (XIII). Comparable to the aluminum surface image of the two strains, both, damaged and dead cells (XIV), as well as seemingly intact cells (XV) were observed.

### 3.5 Copper tolerance and defense related genes in *S. capitis* and *B. lata*

To explain the higher susceptibility of *B. lata* for copper related stress compared to *S. capitis*, genes that are related to copper tolerance or defense, and genes known for coping with oxidative stress were identified in both strains’ genomes (Table 3). Multiple genes were detected in both strains. Surprisingly, more potential copper and oxidative stress response genes were detected in *B. lata* than in *S. capitis*. It should be noted, that the presence of genes in the genome alone does not guarantee their expression. It is, therefore, difficult to make strong interpretations based on this information. Nevertheless, as for *S. capitis*, the copper-sensing transcriptional operon repressor gene *csoR*, has been identified. This gene represents one of the main copper regulators in Gram-positive bacteria (Baker et al., 2011).

**TABLE 3:** Copper tolerance and oxide stress related genes and functions found in the genomes of *S. capitis* and *B. lata.*

Gene	Protein function	*S. capitis*	*B. lata*
*copC*	copper resistance protein; copper homeostasis periplasmic binding protein	×	×
*pcoD*	copper resistance protein D	–	×
*copD*	located in the inner membrane together with pcoD with yet unclear function	–	×
*copZ*	copper chaperone; copper chaperone & heavy metal transport/detoxification protein	×	×
*cycoC*	Heme/copper-type cytochrome/quinol oxidase subunit 3-like	–	×
*cirA*	TonB-dependent copper receptor	–	×
*ompR*	Two-component system copper resistance phosphate regulon response regulator	–	×
*cueR*	Cu(I)-responsive transcriptional regulator	–	×
*soxR*	redox-sensitive transcriptional activator SoxR	–	×
*csoR*	copper-sensing transcriptional repressor	×	–
*csoZ*	putative copper chaperone	×	–
	Copper binding protein	–	×
	Copper transporter	–	×
*sufI*	Multicopper oxidase, type 3	–	×
*sodC*	superoxide dismutase [Cu-Zn]	×	×
	catalase	×	×
*katG, katE, btuE*	Glutathione peroxidase	–	×
	Glutathione peroxidase	×	–
*gstA, yfcF*	Glutathione-S-transferase, glutathione transferase	–	×

### 3.6 Copper ion content in the cell solution after wet contact killing

**FIGURE 6:**
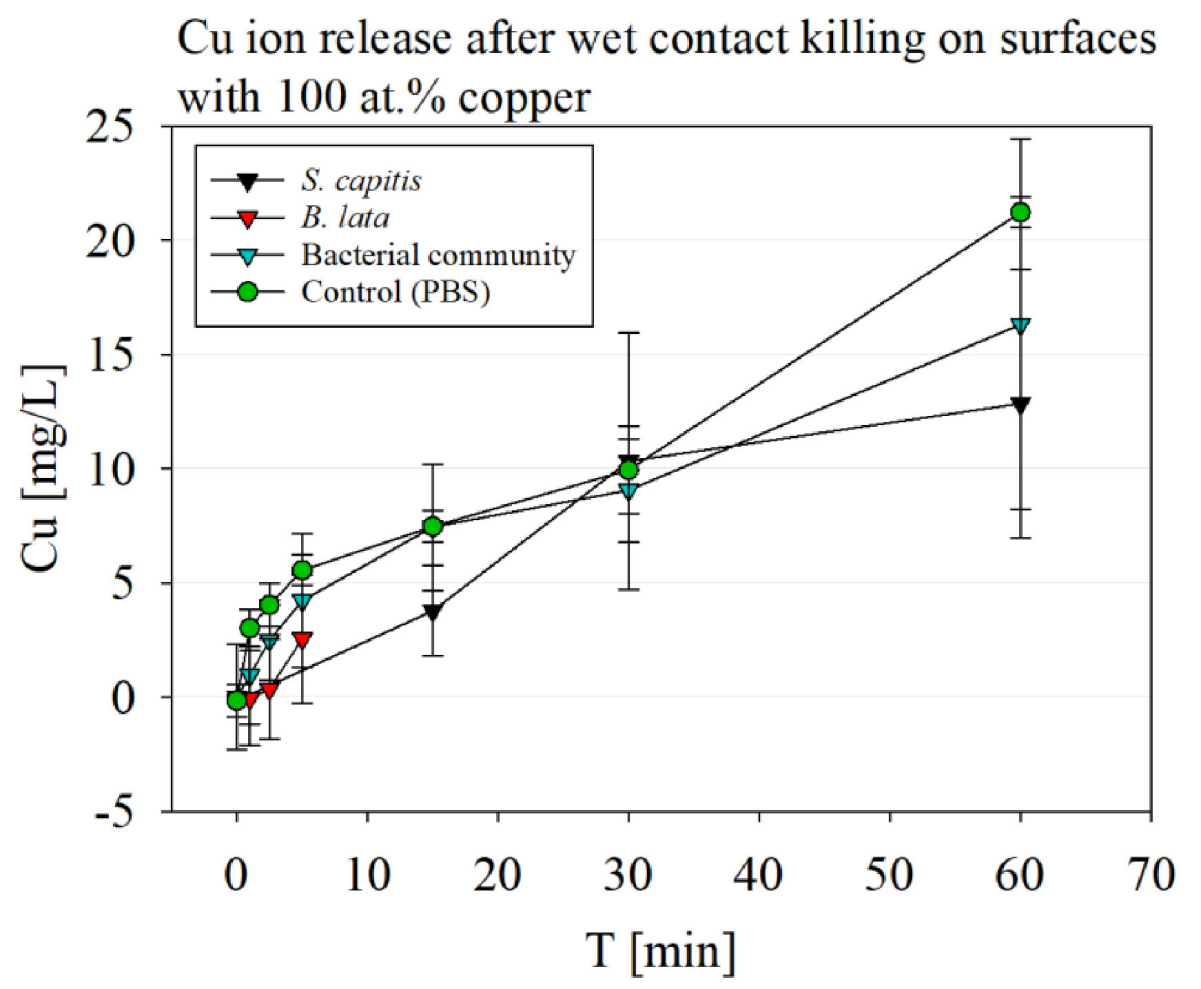
Copper ion release detection via ICP-MS after wet contact killing on surfaces with 100 at.% Cu content coatings, testing *S. capitis*, *B. lata*, bacterial community and the control containing only PBS buffer.

The copper ion release is relevant for the contact killing effectivity. The results of the copper ion release after different time periods of wet contact killing on the 100 at.% Cu surface coating is displayed in Figure 6. Overall, the highest copper ion concentrations were reached in the control samples, containing no bacteria and only PBS (buffer), with 21.22 mg/L after 60 min on the surface.

The copper ion release after wet contact killing with *S. capitis* over a course of 60 min showed overall high standard deviations. The first time point of 15 min pended slightly lower than 5 mg/L, and increased to a similar copper-ion concentration compared to the buffer control to 10 mg/L at 30 min. After 60 min, the copper ion concentration reached ∼13 mg/L.

For *B. lata* samples, only a slight increase of copper-ions was observed after 5 min of wet contact killing, but no significance was determined.

The copper ion release of the surfaces after exposure of the bacterial community to the surfaces over 60 min showed similar concentrations compared to the control, while exhibiting high standard deviations.

The results showed that highest copper ion release was detected in the control solution, PBS. PBS is a salt containing solution, which leads to a corrosion effect, triggering an increased ion release (Luo et al., 2019). Although the copper ion concentration was highest for the PBS control, no significant difference was determined by paired *t*-test compared to the bacterial samples.

## 4 Discussion

In this study, bacterial species were exposed to surface coatings which contained different copper concentrations and aluminum as control element. The tested microorganisms were *S. capitis* DSM 111179, *B. lata* DSM 23089^T^ and a bacterial community containing the two strains mentioned above and seven other species whose genera are most abundant in public transport environments. These species were selected due to the relevance of reducing microbial spread in public transportation environments, where many people come into contact with and transmit microorganisms. Since humans shape an environments’ microbiome, indoor environments have many similarities, as typical skin microorganisms. The microbial composition of an environment can be influenced by surface materials, (Hsu et al., 2016) as well as temperature, relative humidity, and ventilation (Rai et al., 2021). Although the microbial communities vary within different environments, such as public transportation, a core microbiome of the public transport exists which differs to other distinct environments (Danko et al., 2021). Indoor settings such as offices are more likely to have a lower outdoor/indoor exchange compared to mass transit, where doors open every few minutes. Commuters lead to a higher number of people and more variable composition in the cabin environment compared to other indoor settings.

The investigation of the public transport microbiome and the microbial reduction in these settings is relevant for groups of the population that are susceptible to (opportunistic) pathogens, such as elderly and other immune compromised patients. The performed experimental design was carried out to test the difference between the effectiveness of antimicrobial properties against single organisms and organisms within a bacterial community, with the latter closely mimicking real environments. The second aim of the study was to investigate the effectivity of novel surface coatings as a sustainable alternative to full copper materials to investigate the minimal copper concentration for antibacterial effects for more economical and efficient usage.

### 4.1 100 at.% Cu coatings show highest antibacterial activity

The results of the bacterial survival after wet contact killing showed, that only the 100 at.% Cu coating had significant antibacterial effect on both tested strains, as single species and in the bacterial community. The 79 at.% Cu coating only affected the *S. capitis* as single species significantly more than within the bacterial community, but not compared to the control 100 at.% Al. In contrast, previous studies showed that copper alloys with ≥ 58% copper content showed microbial reduction on surfaces (Karpanen et al., 2012). However, the tested surfaces in the study by Karpanen et al. (2012) were investigated over a course of 12 weeks in a real setting with no defined bacterial inoculum, and the overall study design and aims differed. Therefore, it would be of interest to expose cells on the different surfaces used in our study over a longer time period. Another approach to test the antibacterial activity of the surfaces would be dry contact killing, which is closer to real environment settings and has shown to be more effective (Santo et al., 2011). This could be explained by fast copper intake which leads to high copper susceptibility. Furthermore, the bacteria within our study were harvested at the stationary growth phase. Consequently, it would be of interest to perform the wet contact killing experiments with bacteria within the exponential growth phase, since a higher level of sensitivity is expected (Battesti et al., 2011).

In a study by Fowler et al. (2019), the minimum inhibitory concentrations (MIC) of Cu ions were investigated on *Staphylococcus epidermidis*, showing an inhibitory effect after 5 h of exposure to 90 mg/L Cu ions within the sample solution. This result was not observed within the experimental setup of our study. However, both study designs differed. Fowler et al. (2019) conducted direct treatment of Cu ions in culture media, while the bacteria in our study were in PBS, aggravating the Cu ion stress tolerance. We measured Cu ion concentrations between ∼1 and 21 mg/L with bacterial exposure times of 1.0–60 min on 100 at.% Cu. It is noticeable, that the measured Cu ion concentrations were higher compared to other wet contact killing studies, which measured maximum Cu ion concentrations of ∼20 μg/L after 60 min of wet contact killing range (Timofeev et al., 2025). Whereas in our study, Cu ion concentrations reached 21 mg/L. The sample preparation was similar, although different organisms and materials (full copper, no coatings) were used in the study by Timofeev et al. (2025). Interestingly, the detected Cu ion concentrations are more comparable to a wet contact killing study, which was conducted with *Escherichia coli* K12 (Luo et al., 2017).

It shows, that Cu ions seem to have a different weight of effect on the different bacterial species, which can be attributed to the variability of copper defense mechanisms and their effectivity, as described in following discussion (Solioz et al., 2010; Bondarczuk and Piotrowska-Seget, 2013; Giachino and Waldrom, 2020; Lima et al., 2022).

The decreased antibacterial effect of the Cu-Al alloy coatings could be traced back to the significantly lower Cu ion release of the alloys compared to the 100 at.% Cu coatings (Supplementary Figure 4). The reason for the lower Cu ion release could be related to the intermetallic phases between Cu and Al, which are strongly bound and allows for only reduced Cu ion release (Shi et al., 2020). For more detailed investigation of the antibacterial properties of the surfaces, a closer look to oxidative stress caused by ROS will be helpful. This could be performed indirectly by the investigation of possible protein oxidation or DNA damage using gel electrophoresis, or membrane damage could be observed by fluorescence microscopy and LIVE/DEAD staining (Fernández et al., 2011; Berney et al., 2007). A direct detection of ROS could be conducted using oxidative stress markers (Li et al., 2016).

### 4.2 Different genotypes result in different survival

The wet contact killing experiments showed that both tested bacterial strains were not similar in their susceptibility to copper. Although *B. lata* was exposed to an overall low copper ion concentration of 5 mg/L after 5 min, its susceptibility to the 100 at.% copper containing surface was significantly higher with a > 4-log reduction in survival, compared to *S. capitis*, which was exposed to ∼13 mg/L after 60 min. The survival of *S. capitis* after 15 min of wet contact killing was similar to the survival of *B. lata* after only 2.5 min of wet contact killing on 100 at.% copper content surface. The high susceptibility of *B. lata* to copper was already observed in preliminary tests, exhibiting a 100% reduction after only 15 min of exposure (data not shown). This observation is not reflected in studies that have tested different strains of *Burkholderia glumae.* In a study which conducted contact killing assay with strains of *Burkholderia glumae*, survival was still detectable after 4 h of contact time (Cui et al., 2014). However, membrane damage was already observed after 1 min of contact, visualized by LIVE/DEAD staining. This was not clearly shown in our study by SEM of *B. lata* after 5 min of contact, indicating a high variability of behavior even among the same genus. A reason for this discrepancy related to copper tolerance could be manifold. Different species can differ drastically due to their different isolation and genotypes. The isolation sides differ between *B. glumae* (rice seeds) and *B. lata* (forest soil) and therefore selective pressure led to a different set of genes. Additionally, the genome sizes of *B. glumae* LMG 2196 with 6.8 Mbp and *B. glumae* AU6208 with 6.1 Mbp are smaller compared to *B. lata* with 8.6 Mbp allowing a greater gene number. While the authors found *copC* in the tested strains of *B. glumae*, we found multiple genes related to copper tolerance and defense in *B. lata* and *S. capitis*. Nevertheless, *B. glumae* equipped with only *copC* lead to a higher copper tolerance compared to *B. lata.* It is remarkable, that more copper resistance-related genes are encoded in *B. lata* compared to *B. glumae* and *S. capitis* despite its decreased survival after copper contact. The genes found in *B. lata* were e.g. *copC, copD, copZ, pcoD, sufI*, and *cirA* and in *S. capitis*, relevant genes such as *copC, csoZ*, *copZ*, and *csoR* were found. However, the actual expression of those genes is unclear and needs to be investigated in future studies. Further, the function of these genes can differ between genera. Consequently, the function of those genes can only be assumed based on available findings that investigated other bacterial species and genera. The occurrence of copper- and oxidative stress related genes in both *B. lata* and *S. capitis* was compared with existing data on similar species, if information was available. For *B. lata*, the gene *copC*, coding for the copper resistance protein or copper homeostasis periplasmic binding protein was detected. It has been shown on *Burkholderia cenocepacia*, that copC functions as a Cu ion binding protein, allowing copper detoxification (Higgins et al., 2020). *copC* was also detected in *Staphylococcus aureus*, acting as a copper transport gene (Kinnevey et al., 2013). Other genes that were detected in the tested *S. capitis* such as *csoZ*, *copZ*, and *csoR* were found in *S. aureus* before (Baker et al., 2011). The *csoR* gene, encoding for the copper-sensing transcriptional repressor, is considered as one of the main copper regulators in Gram-positive bacteria (Baker et al., 2011), which we assume to be significant in the great survival of *S. capitis* compared to *B. lata.*

### 4.3 Gram-negative vs. gram-positive bacteria in their copper defense

As shown above, even species of one genus can differ in their survivability against antibacterial copper surfaces. What distinguishes *B. lata* from *S. capitis*, is their gram stain and therefore their bacterial structure. *S. capitis* is gram-positive and *B. lata* is gram-negative. Other gram-negative strains such as the model organism *Escherichia coli* or *Pseudomonas aeruginosa* have been tested in previous studies on surfaces with different copper contents. *E. coli* showed total inactivation varying between 15 and 180 min (Wilks et al., 2005; Różańska et al., 2017). *P. aeruginosa* showed an inhibition of 99.9% when treated with 800 μg/mL of copper nanoparticles, while *S. capitis* could endure this concentration (Betancourt-Galindo et al., 2014). The difficulty of comparing study designs and model organisms is highlighted by the observed differences in antimicrobial effectivity of surfaces across studies. The high survival of *S. capitis* was anticipated due to a thick layer of peptidoglycan, which has been suggested to be the reason for better survival compared to gram-negative *Pseudomonas aeruginosa* after treatment with copper nanoparticles (Betancourt-Galindo et al., 2014). Studies such as Różańska et al. (2017) showed, similar to our results, that *Staphylococcus* survived on surfaces for long periods of time, ranging from ∼45 to ∼220 min, depending on the copper alloys tested. Although both gram-positive (*S. capitis*) and gram-negative (*B. lata*) bacteria were susceptible to copper toxicity, the gram-negative strain used in our study showed significantly higher susceptibility, which may be due to increased lipid peroxidation, leading to membrane damage, DNA damage and cell death (San et al., 2015). Further, the above-mentioned genes related to copper tolerance and defense could be higher regulated in *S. capitis* compared to *B. lata*. This has to be investigated in future studies. Nevertheless, gram-negative bacteria are not expected to be more susceptible to copper stress compared to gram-positive in general. Gram-negative bacteria are able to provide active control through the outer- and inner membrane and are equipped with a strong efflux pump system (Bondarczuk and Piotrowska-Seget, 2013). Under consideration of the cell sizes (*S. capitis* 1 μm, coccoid; *B. lata* 2 μm, rod-shaped), 83.4% of 10^7^ cells of *S. capitis* were expected to occur as a monolayer on the 6 mm diameter surface contact area, while 89.4% were calculated for *B. lata*. This could enhance the higher susceptibility of *B. lata* to copper compared to *S. capitis*, but only to some extent, since the calculations are based on one selected size and without taking the cell shape into account. However, the cell size varies, e.g., it can be between 0.5 and 1.0 μm for *S. capitis*.

### 4.4 Bacterial survival after long exposure times is higher within a bacterial community

As shown by Salah et al. (2021), many studies have tested the antimicrobial effectiveness of different copper and copper alloy materials and surfaces. However, while most studies were conducted with strains of *E. coli* or *S. aureus*, none of them tested the effectiveness against microbial communities. In this study, we tested a bacterial consortium of nine bacterial strains with different genera, based on Ly et al. (2024). The results of this work showed the reduced antibacterial effectivity of the test surfaces on the bacterial community in comparison to single species.

With long term use of antimicrobial materials, it should be noted that the microbiome of surfaces change. The composition can change and the diversity has been observed to lower within microbial communities that have been exposed to increased copper concentrations (Schmidt et al., 2012; Sutcliffe et al., 2019; Shaw et al., 2020). Occurring species that were copper sensitive decreased, and copper tolerant species occurred to be more dominant in the microbiome (Gustavson et al., 1999; Serra et al., 2009; Ancion et al., 2010; Corcoll et al., 2019). These studies did not investigate associated resistance to antibiotics, although it displays an important aspect that was shown in a study by Arndt et al. (2025, unpublished manuscript). The authors demonstrated the risk of antibiotic resistance enhancing the copper tolerance in strains of *Enterococcus faecium*, highlighting the conscious application of countermeasures and drug use. This specific combination of antibiotic resistance and surface transmission has been subject of many studies within the clinical environment (Stephens et al., 2019; Xiao et al., 2019). However, the transmission of (opportunistic) pathogens is not limited to the clinical environment, but is relevant for all public environments, where the spread and emergence of antibiotic resistances needs to be reduced.

Within our used study design, cell layering on the surface was expected and intended for a more realistic approach. The determination of the actual multilayer was a challenge within this study, since the different cell sizes and shapes vary enormously and therefore error would be expected. One hypothesis for the increased survival of the selected species *B. lata* and especially *S. capitis* within the bacterial community is the cell layering and reduced contact to the surfaces. Due to varying cell morphologies of the different used bacterial species (as shown in see section “3.4 SEM imaging of bacterial cells on copper and aluminum containing coatings”), layers could be thicker than those resulting from layering of single species. Thus, less cells of *S. capitis* and *B. lata* were exposed to the surfaces when the bacterial community was tested. As a result, less cells could be damaged.

Another hypothesis is based on the metabolic interaction between the bacterial species. Metabolic interaction can lead to the spread and use of metabolic compounds that help cells which are normally more susceptible to copper (Williams et al., 2016). Copper defense mechanisms by bacteria within the bacterial community, such as the production of extracellular vesicles to secrete copper out of cells, could also benefit other bacterial species due to a lower toxic copper concentration in the environment (Lima et al., 2022). Inter-bacterial interactions and metabolic changes are already known among bacteria against toxic compounds (Hong et al., 2018; Dai et al., 2024). Since the survival of *B. lata* within the bacterial community was higher compared to the single exposure of *B. lata* to copper even after only 5 min of contact, rapid metabolic changes and inter-bacterial interactions could have impacted the increased survival besides the cell layering as described in the previous section.

The formation of biofilms is a common structure which leads to the resistance of bacterial cells against antibacterial compounds. However, this is not expected for the experiments, since the cells were present in PBS, a low nutrient solution. Therefore, potential biofilm formation by e.g., biofilm forming species *S. capitis* was not expected for the copper exposure times of up to 5 and 60 min, respectively.

### 4.5 Metabolic activity after surface exposure corresponds with bacterial survival

After wet contact killing on 100 at.% copper surface, the metabolism of both species *S. capitis* and *B. lata* showed significantly reduced and delayed activity in both scenarios as single species and within the bacterial community corresponding with results of their respective bacterial survival. The single species testing showed reduced metabolic activity compared to the bacterial community, especially for *B. lata*. Here, even the 79 at.% copper surface caused a delayed metabolic activity of the cells, although it was not reflected in the survival data. This could be explained by the strategic metabolic downregulation to preserve important functions for survival while minimizing cell damage (Traxler et al., 2011; Qi et al., 2023). However, a decrease would also be observed in the survival, which was not the case in our study. Another mechanism which maintains survivability and simultaneously alters the metabolic activity of bacterial cells is antioxidant response. The oxidative stress that is induced by copper might have caused a temporary metabolic downregulation and, therefore, delay of the alamarBlue metabolization. Simultaneously, oxidative stress response would activate the production of antioxidants, such as glutathione peroxidase, superoxide dismutase and catalase, which are genes found in *S. capitis* and *B. lata*. Beside the oxidative stress response, multiple genes related to copper defense that were found in both genomes, such as *copC* and *copZ* are assumed to be upregulated during copper stress, as well as active copper transport (Teitzel et al., 2006). The *cirA* gene was found in *B. lata*, and is evidently downregulated in *Acidithiobacillus ferrooxidans* D2 during copper stress. *cirA* is a TonB-dependent copper receptor, which restricts copper uptake by the regulation of transporters (Salazar et al., 2013).

A clear determination and overview of genes, which are up- and downregulated during copper stress in the tested species within this study is not given and, therefore, poses a limitation of this study. Nevertheless, we assume that the metabolic upregulation of genes related to copper stress was not significant, especially in *B. lata* due to its high susceptibility to the wet contact killing assay, which was reflected in the reduced metabolic activity on the 79 at.% Cu and 100 at.% Cu surfaces. In general, the metabolic activity assay confirmed the survival data, and showed additional susceptibility of the tested strains toward exposure to copper surfaces that was not visible by bacterial survival alone.

### 4.6 Intact cell morphology after copper exposure

The SEM imaging of the 100 at.% copper and 100 at.% aluminum coatings after contact killing, showed a slight increase of extracellular products and other organic matrix surrounding the cells on copper surfaces. Those accumulations could be determined as extracellular vesicles, which form after stress response and have been observed both in gram-positive and gram-negative bacteria (Kim et al., 2015; Bose et al., 2020; Briaud and Carroll, 2020). Such structures have been shown in a cyanobacterial strain and were identified as a copper-secretion mechanism (Lima et al., 2022). Especially for *S. capitis*, the accumulation of the vesicles was increased on the copper surfaces, indicating the stress response. EDS analysis of the material showed occurrence of sulfur (Supplementary Figure 5), which has been observed in extracellular vesicles before (Noundou et al., 2024). However, sulfur containing extracellular vesicles are rare. Therefore, a likely possibility would be that the accumulations are copper oxides, as shown in Akintelu et al. (2020).

The cell morphology showed only little visual damage, or no significant damage compared to the cells observed on the 100 at.% Al coated surface, which may be attributed to the multilayer of cells on the surface. This observation should be deeper investigated in future studies. Contrary to these results, cell disruption was clearly observed in a study by Gopinath et al. (2016) after 60 min exposure to CuO nanoparticles. A reason for the observed cell disruption could be due to the exposure to the bacterial species MIC concentrations of the CuO nanoparticles, whereas in our study, no defined copper concentration but rather time was set. Further, the authors study conducted experiments in CuO nanoparticle solution, in contrast to our study on surfaces.

### 4.7 Potential for further enhancements of copper coatings

So far, various studies have been conducted with copper alloys. We created and tested Cu-Al-coatings as a feasible but more sustainable approach. To our dissatisfaction, only 100 at.% Cu coatings were sufficient for bacterial reduction. However, we were able to identify options for improvements: Firstly, another metal than aluminum could be used to create copper alloys, since it is a rather soft material.

Next, the coatings were generated by magnetron sputtering. This is a well-established method for creating coatings, such as implant coatings (Tian et al., 2007; Ferraris and Spriano, 2016). However, intermetallic phases formed during the deposition of the Cu-Al alloys. Since Cu-atoms are rigidly situated within these structures, Cu ions are less likely to be released (Shi et al., 2020). This is in accordance with our Cu ion content measurement of the PBS and bacterial solution after wet contact killing on Cu-Al alloy samples (Supplementary Figure 4), which is most likely contributing to the lower antibacterial effect, as described in see section “4.4 Bacterial survival after long exposure times is higher within a bacterial community.” Another alloy of interest would be Al-Ag-Cu, which has shown to have a greater antibacterial effect compared to Cu-Al alloys on strains of *E. coli*, explained by the higher proportion of antimicrobial metals within the Al phase (Hahn et al., 2018). However, since aluminum proposes a rather soft material which is prone to damage, other metals such as zinc (Almoudi et al., 2018) could be used.

The durability and stability under real-life conditions of the coatings have to be addressed in future studies, as the focus of this study was on the antibacterial activity and effectivity. Glass slides were purposely selected as substrate material, since they compose of standardized roughness and therefore support reproducibility of the results. Therefore, the mechanical and tribological properties of the coatings should be studied as well as corrosion under conditions involving cleaning agents, operational environments, and microbial biofilms.

## 5 Conclusion and outlook

In this study, the bacterial community showed greater survival after exposure to the Cu and Cu-Al coatings, demonstrating the importance of testing not only single species. The coatings showed highest antibacterial activity when the Cu content was 100 at.%. Metabolic activity measurements supported the bacterial survival data.

Scanning electron microscope imaging revealed that cells exhibited morphological damage on the 100 at.% Cu coatings, although this damage was not differing significantly from the 100 at.% Al coatings after 1–2 h of contact time. While more genes related to copper and oxidative stress defense were found in *B. lata*, survival of *S. capitis* was significantly higher. This observation will be focused on in future studies by investigating the gene expression.

The copper ion content following wet contact killing on 100 at.% Cu coatings was comparatively high compared to other studies, but could be explained by the coatings showing early degradation under the SEM. However, Cu-Al alloy coatings exhibited significantly reduced copper ion release compared to the 100 at.% Cu coating. This phenomenon can be attributed to the formation of a robust intermetallic phase between Cu and Al, which hinders ion release, explaining the weak antibacterial activity of these alloy coatings.

In subsequent studies, a more thorough examination of the wet contact killing process, as well as the dry contact killing process, could provide additional information about the test materials and their antibacterial activity. An additional approach would be to investigate the expression of copper tolerance or copper defense related genes to better understand the differential survival of species within the bacterial community. These approaches can be evaluated using current methodologies and also with new synthesized surface coatings incorporating other metals.

To conclude, this study provided new findings on wet contact killing effects on bacteria, both as pure culture and as bacterial community. Additionally, novel copper-based surface coatings were tested and will be improved in future investigations. Our study on this topic contributes to the understanding of copper as an antibacterial countermeasure, with the aim to combat the transmission of opportunistic bacteria that pose a human health risk.

## Data Availability

The datasets presented in this study can be found in online repositories. The names of the repository/repositories and accession number(s) can be found below: https://www.ncbi.nlm.nih.gov/, PRJNA1283126.

## Footnotes

1 Westmetall (2024). Marktdaten [Online]. Available: https://www.westmetall.com/de/markdaten.php [Accessed 23.09.2024].

2 ThermoFisher Scientific (2025). alamarBlue Assays for Cell Viability Protocol, for Microplates [Online]. Available: https://www.thermofisher.com/de/de/home/references/protocols/cell-and-tissue-analysis/protocols/alamarblue-cell-viability-reagent-for-microplates-protocol.html [Accessed 04-15 2025].

3 https://support.illumina.com/content/dam/illumina-support/documents/documentation/chemistry_documentation/samplepreps_truseq/truseqnanodna/truseq-nano-dna-library-prep-guide-15041110-d.pdf [Accessed 07-23 2025].
