# Supplementary material for: Comparison of single bacteria and a bacterial reference community in a test against coated surfaces of varying copper content
Source: Front Microbiol. 2025 Aug 26;16:1659828. doi: 10.3389/fmicb.2025.1659828 (PMC12417388; doi:10.3389/fmicb.2025.1659828)
Supplement: Supplementary file 1 [file Data_Sheet_1.docx]

**Supplementary Material**

**Table S1:** CFU/mL of *B. lata* as pure culture and within the bacterial community after wet contact killing.

| **Surface** | **Contact time** | ***B. lata*:**  **Mean CFU/mL** | **SD (CFU/mL)** | ***B. lata* in community:**  **Mean CFU/mL** | **SD (CFU/mL)** |
| --- | --- | --- | --- | --- | --- |
|  | 0.0 min | 2.13 × 10^8^ | 1.03 × 10^8^ | 2,27 × 10^7^ | 9.29 × 10^6^ |
| 100 at.% Al | 1.0 min | 1.56 × 10^8^ | 6.90 × 10^7^ | 2.19 × 10^7^ | 1.14 × 10^7^ |
|  | 2.5 min | 1.69 × 10^8^ | 6.81 × 10^7^ | 2.20 × 10^7^ | 7.44 × 10^6^ |
|  | 5.0 min | 1.60 × 10^8^ | 7.53 × 10^7^ | 1.88 × 10^7^ | 7.01 × 10^6^ |
| 100 at.% Cu | 1.0 min | 3.79 × 10^7^ | 3.40 × 10^7^ | 3.79 × 10^6^ | 4.67 × 10^6^ |
|  | 2.5 min | 1.96 × 10^6^ | 2.41 × 10^6^ | 2.23 × 10^5^ | 3.14 × 10^5^ |
|  | 5.0 min | 7.40 × 10^3^ | 1.14 × 10^4^ | 5.72 × 10^4^ | 8.09× 10^4^ |
| 79 at.% Cu | 1.0 min | 8.57 × 10^7^ | 6.01 × 10^7^ | 2.33 × 10^7^ | 8.50× 10^5^ |
|  | 2.5 min | 1.13 × 10^8^ | 7.98 × 10^7^ | 2.52 × 10^7^ | 3.57 × 10^6^ |
|  | 5.0 min | 1.44 × 10^8^ | 1.12 × 10^8^ | 2.33 × 10^7^ | 1.31 × 10^6^ |
| 53 at.% Cu | 1.0 min | 1.49 × 10^8^ | 6.19 × 10^7^ | 3.53 × 10^7^ | 5.19 × 10^6^ |
|  | 2.5 min | 9.71 × 10^7^ | 4.58 × 10^7^ | 3.65 × 10^7^ | 5.10 × 10^6^ |
|  | 5.0 min | 1.05 × 10^8^ | 7.02 × 10^7^ | 3.35 × 10^7^ | 5.12 × 10^6^ |
| 24 at.% Cu | 1.0 min | 1.67 × 10^8^ | 5.39 × 10^7^ | - | - |
|  | 2.5 min | 1.72 × 10^8^ | 8.27 × 10^6^ | - | - |
|  | 5.0 min | 1.72 × 10^8^ | 1.53 × 10^7^ | - | - |

**Table S2:** CFU/mL of *S. capitis* as pure culture and within the bacterial community after wet contact killing.

| **Surface** | **Contact**  **time** | ***S. capitis*:**  **Mean CFU/mL** | **SD (CFU/mL)** | ***S. capitis* in community:**  **Mean CFU/mL** | **SD (CFU/mL)** |
| --- | --- | --- | --- | --- | --- |
|  | 0.0 min | 6.64 × 10^8^ | 1.04 × 10^9^ | 3.57 × 10^7^ | 1.01 × 10^7^ |
| 100 at.% Al | 15 min | 2.01 × 10^8^ | 5.33 × 10^7^ | 3.13 × 10^7^ | 3.46 × 10^6^ |
|  | 30 min | 1.83 × 10^8^ | 1.03 × 10^8^ | 3.29 × 10^7^ | 4.56 × 10^6^ |
|  | 60 min | 2.33 × 10^8^ | 6.65 × 10^7^ | 3.50 × 10^7^ | 9.05 × 10^6^ |
| 100 at.% Cu | 15 min | 5.18 × 10^7^ | 7.13 × 10^7^ | 7.25 × 10^6^ | 9.61 × 10^6^ |
|  | 30 min | 7.64 × 10^6^ | 1.19 × 10^7^ | 6.71 × 10^5^ | 9.39 × 10^5^ |
|  | 60 min | 2.21 × 10^3^ | 3.82 × 10^3^ | 5.56 × 10^2^ | 4.16 × 10^2^ |
| 79 at.% Cu | 15 min | 1.44 × 10^8^ | 7.34 × 10^7^ | 3.32 × 10^7^ | 4.48 × 10^6^ |
|  | 30 min | 1.05 × 10^8^ | 7.34 × 10^7^ | 6.80 × 10^7^ | 4.44 × 10^7^ |
|  | 60 min | 8.66 × 10^7^ | 8.95 × 10^7^ | 4.94 × 10^7^ | 6.35 × 10^6^ |
| 53 at.% Cu | 15 min | 1.92 × 10^8^ | 2.80 × 10^7^ | 3.78 × 10^7^ | 3.01 × 10^6^ |
|  | 30 min | 5.64 × 10^8^ | 5.08 × 10^8^ | 3.18 × 10^7^ | 7.73 × 10^6^ |
|  | 60 min | 1.71 × 10^8^ | 1.13 × 10^8^ | 3.28 × 10^7^ | 1.31 × 10^6^ |
| 24 at.% Cu | 15 min | 2.02 × 10^8^ | 4.66 × 10^7^ | - | - |
|  | 30 min | 1.60 × 10^8^ | 1.91 × 10^7^ | - | - |
|  | 60 min | 2.23 × 10^8^ | 5.95 × 10^7^ | - | - |

**Table S3:** Survival fraction N/N_0_ mean values of *B. lata* as single species or within the bacterial community after wet contact killing.

| **Surface** | **Contact time** | ***B. lata*:**  **Mean N/N_0_** | **SD (N/N_0_)** | ***B. lata* in community:**  **Mean N/N_0_** | **SD (N/N_0_)** |
| --- | --- | --- | --- | --- | --- |
| 100 at.% Al | 1.0 min | 8.44 × 10^-1^ | 4.14 × 10^-1^ | 9.77 × 10^-1^ | 2.57 × 10^-1^ |
|  | 2.5 min | 8.95 × 10^-1^ | 4.07 × 10^-1^ | 1.00 × 10^0^ | 1.72 × 10^-1^ |
|  | 5.0 min | 8.53 × 10^-1^ | 3.59 × 10^-1^ | 8.47 × 10^-1^ | 2.02 × 10^-1^ |
| 100 at.% Cu | 1.0 min | 2.19 × 10^-1^ | 2.40 × 10^-1^ | 2.05 × 10^-1^ | 4.82 × 10^-1^ |
|  | 2.5 min | 1.20 × 10^-2^ | 2.29 × 10^-2^ | 2.07 × 10^-2^ | 3.53 × 10^-2^ |
|  | 5.0 min | 3.74 × 10^-5^ | 7.71 × 10^-5^ | 5.33 × 10^-3^ | 9.20 × 10^-3^ |
| 79 at.% Cu | 1.0 min | 4.84 × 10^-1^ | 3.64 × 10^-1^ | 9.93 × 10^-1^ | 3.62 × 10^-2^ |
|  | 2.5 min | 6.61 × 10^-1^ | 5.44 × 10^-1^ | 1.07 × 10^0^ | 1.52 × 10^-1^ |
|  | 5.0 min | 8.16 × 10^-1^ | 6.65 × 10^-1^ | 9.93 × 10^-1^ | 5.58 × 10^-2^ |
| 53 at.% Cu | 1.0 min | 8.51 × 10^-1^ | 3.31 × 10^-1^ | 9.47 × 10^-1^ | 1.07 × 10^-1^ |
|  | 2.5 min | 5.66 × 10^-1^ | 2.90 × 10^-1^ | 1.00 × 10^0^ | 1.58 × 10^-1^ |
|  | 5.0 min | 6.01 × 10^-1^ | 4.20 × 10^-1^ | 9.23 × 10^-1^ | 1.22 × 10^-1^ |
| 24 at.% Cu | 1.0 min | 7.78 × 10^-1^ | 4.86 × 10^-1^ | - | - |
|  | 2.5 min | 8.27 × 10^-1^ | 3.69 × 10^-1^ | - | - |
|  | 5.0 min | 8.37 × 10^-1^ | 3.41 × 10^-1^ | - | - |

**Table S4:** Survival fraction N/N_0_ mean values of *S. capitis* single species or within the bacterial community after wet contact killing.

| **Surface** | **Contact time** | ***S. capitis*:**  **Mean N/N_0_** | **SD (N/N_0_)** | ***S. capitis* in community:**  **Mean N/N_0_** | **SD (N/N_0_)** |
| --- | --- | --- | --- | --- | --- |
| 100 at.% Al | 15 min | 7.33 × 10^-1^ | 4.32 × 10^-1^ | 1.05 × 10^0^ | 2.62 × 10^-1^ |
|  | 30 min | 6.89 × 10^-1^ | 3.54 × 10^-1^ | 1.06 × 10^0^ | 2.71 × 10^-1^ |
|  | 60 min | 8.04 × 10^-1^ | 3.60 × 10^-1^ | 1.08 × 10^0^ | 3.26 × 10^-1^ |
| 100 at.% Cu | 15 min | 7.89 × 10^-2^ | 1.03 × 10^-1^ | 6.54 × 10^-2^ | 1.04 × 10^-1^ |
|  | 30 min | 6.86 × 10^-3^ | 7.38 × 10^-3^ | 5.21 × 10^-4^ | 7.23 × 10^-4^ |
|  | 60 min | 6.90 × 10^-7^ | 1.37 × 10^-6^ | 8.82 × 10^-5^ | 1.20 × 10^-4^ |
| 79 at.% Cu | 15 min | 6.63 × 10^-1^ | 5.06 × 10^-1^ | 1.08 × 10^0^ | 1.46 × 10^-1^ |
|  | 30 min | 4.91 × 10^-1^ | 4.02 × 10^-1^ | 2.22 × 10^0^ | 1.45 × 10^0^ |
|  | 60 min | 4.24 × 10^-1^ | 5.02 × 10^-1^ | 1.61 × 10^0^ | 2.07 × 10^-1^ |
| 53 at.% Cu | 15 min | 7.77 × 10^-1^ | 3.60 × 10^-1^ | 1.11 × 10^0^ | 1.24 × 10^-1^ |
|  | 30 min | 2.62 × 10^0^ | 5.31 × 10^0^ | 1.58 × 10^0^ | 1.22 × 10^0^ |
|  | 60 min | 6.85 × 10^-1^ | 5.80 × 10^-1^ | 1.30 × 10^0^ | 3.49 × 10^-1^ |
| 24 at.% Cu | 15 min | 8.95 × 10^-1^ | 2.70 × 10^-1^ | - | - |
|  | 30 min | 6.96 × 10^-1^ | 1.62 × 10^-1^ | - | - |
|  | 60 min | 1.01 × 10^0^ | 5.59 × 10^-1^ | - | - |

**Table S5:** p-values of t-tests of single species after wet contact killing of survival fraction 100 at.% Al vs. other surfaces types. Contact time 1 min, 2) 2.5 min, 3) 5 min. Contact time *S. capitis* 1) 15 min, 2) 30 min, 3) 60 min.

| **Surface** | **Contact time** | ***B. lata* single**  **(p-value)** | **Significance** | ***S. capitis* single**  **(p-value)** | **Significance** |
| --- | --- | --- | --- | --- | --- |
| 100 at.% Cu | 1 | <0.001 | *** | <0.001 | *** |
|  | 2 | <0.001 | *** | <0.001 | *** |
|  | 3 | <0.001 | *** | <0.001 | *** |
| 79 at.% Cu | 1 | 0.099 | ns | 0.580 | ns |
|  | 2 | 0.512 | ns | 0.252 | ns |
|  | 3 | 0.884 | ns | 0.076 | ns |
| 53 at.% Cu | 1 | 0.734 | ns | 0.976 | ns |
|  | 2 | 0.060 | ns | 0.504 | ns |
|  | 3 | 0.118 | ns | 0.521 | ns |
|  | 1 | 0.319 | ns | 0.505 | ns |
| 24 at.% Cu | 2 | 0.803 | ns | 0.959 | ns |
|  | 3 | 0.916 | ns | 0.959 | ns |

**Table S6:** p-values of t-tests of species within the bacterial community after wet contact killing of survival fraction 100 at.% Al vs. other surfaces types. Contact time 1 min, 2) 2.5 min, 3) 5 min. Contact time *S. capitis* 1) 15 min, 2) 30 min, 3) 60 min.

| **Surface** | **Contact time** | ***B. lata* in community**  **(p-value)** | **Significance** | ***S. capitis* in community**  **(p-value)** | **Significance** |
| --- | --- | --- | --- | --- | --- |
| 100 at.% Cu | 1 | 0.001 | *** | <0.001 | *** |
|  | 2 | <0.001 | *** | <0.001 | *** |
|  | 3 | <0.001 | *** | <0.001 | *** |
| 79 at.% Cu | 1 | 0.818 | ns | 0.687 | ns |
|  | 2 | 0.586 | ns | 0.159 | ns |
|  | 3 | 0.070 | ns | 0.043 | * |
| 53 at.% Cu | 1 | 0.701 | ns | 0.571 | ns |
|  | 2 | 0.994 | ns | 0.594 | ns |
|  | 3 | 0.319 | ns | 0.250 | ns |

**Table S7:** p-values of t-tests. Contact time for *B. lata* 1) 1 min, 2) 2.5 min, 3) 5 min. Contact time *S. capitis* 1) 15 min, 2) 30 min, 3) 60 min.

| **Surface** | **Contact time** | ***B. lata*:**  **Single vs. community (p-value)** | **Significance** | ***S. capitis*:**  **Single vs. community**  **(p-value)** | **Significance** |
| --- | --- | --- | --- | --- | --- |
| 100 at.% Cu | 1 | 0.055 | ns | 0.831 | ns |
|  | 2 | 0.176 | ns | 0.125 | ns |
|  | 3 | 0.818 | ns | 0.013 | * |
| 79 at.% Cu | 1 | 0.004 | ** | 0.227 | ns |
|  | 2 | 0.266 | ns | 0.232 | ns |
|  | 3 | 0.478 | ns | 0.021 | * |
| 53 at.% Cu | 1 | 0.469 | ns | 0.038 | * |
|  | 2 | 0.008 | ** | 0.195 | ns |
|  | 3 | 0.069 | ns | 0.051 | ns |

**Table S8:** AlamarBlue assay significance testing. Two-way repeated measures ANOVA testing of positive control vs. single species samples after 5 min (*B. lata*) and 60 min (*S. capitis*) of contact killing.

| **Positive control vs.** | ***B. lata* single**  **(p-value)** | **Significance** | ***S. capitis* single**  **(p-value)** | **Significance** |
| --- | --- | --- | --- | --- |
| 100 at.% Cu | 0.002 | ** | 0.003 | ** |
| 79 at.% Cu | <0.001 | *** | 0.219 | ns |
| 53 at.% Cu | 0.639 | ns | 0.011 | * |
| 24 at.% Cu | 0.312 | ns | 0.024 | * |

**Table S9:** AlamarBlue assay significance testing. Two-way repeated measures ANOVA testing of positive control vs. species within the bacterial community samples after 5 min (*B. lata*) and 60 min (*S. capitis*) of contact killing.

| **Positive control vs.** | ***B. lata* in community**  **(p-value)** | **Significance** | ***S. capitis* in community**  **(p-value)** | **Significance** |
| --- | --- | --- | --- | --- |
| 100 at.% Cu | 0.009 | ** | 0.004 | ** |
| 79 at.% Cu | 0.001 | *** | 0.008 | ** |
| 53 at.% Cu | 0.004 | ** | 0.005 | ** |

**Figure S1** displays the metabolic activity via alamarBlue assay of *S. capitis* after exposure to the surfaces and settings as described earlier. For the 24 at.% (**Figure S1a**) copper coated surface, no antibacterial effect was observed, which correlates with the survival data. Surprisingly, the 53 at.% (**Figure S1b**) copper surface led to a reduction of *S. capitis* metabolic activity after 60 min exposure, which was not seen in the survival data. More noticeable is the effect on the 79 at.% copper content surface (**Figure S1c**). After only 15 min of contact, a great reduction was visible, and after 30 and 60 min, no metabolic activity was observed after 20 h incubation time. Similarly, the survival data did not indicate a deficiency in survival, yet the metabolic activity was observed to be markedly diminished. Unsurprisingly, the exposure of *S. capitis* on surface coatings with 100 at.% copper (**Figure  S1d**) resulted in no significant metabolic activity after 20 h of measurement. Interestingly, the exposure of the cells on Aluminum coatings (**Figure S1e**) resulted in a maximum resazurin reduction of approximately 55 %. In contrast, highest reduction was observed with almost 100 % on 24 at.% copper coatings. This indicates an antimicrobial effect of Aluminum.

**
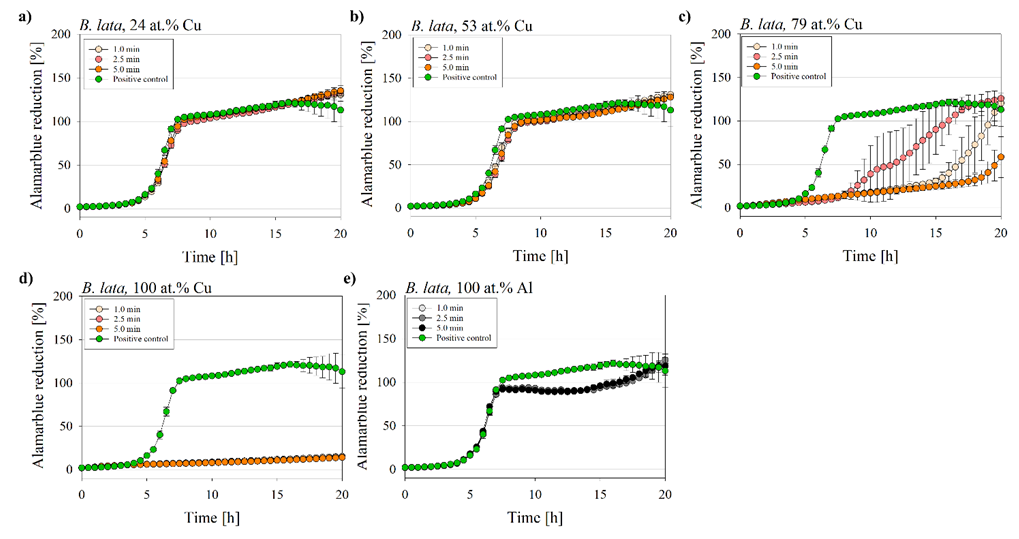
**
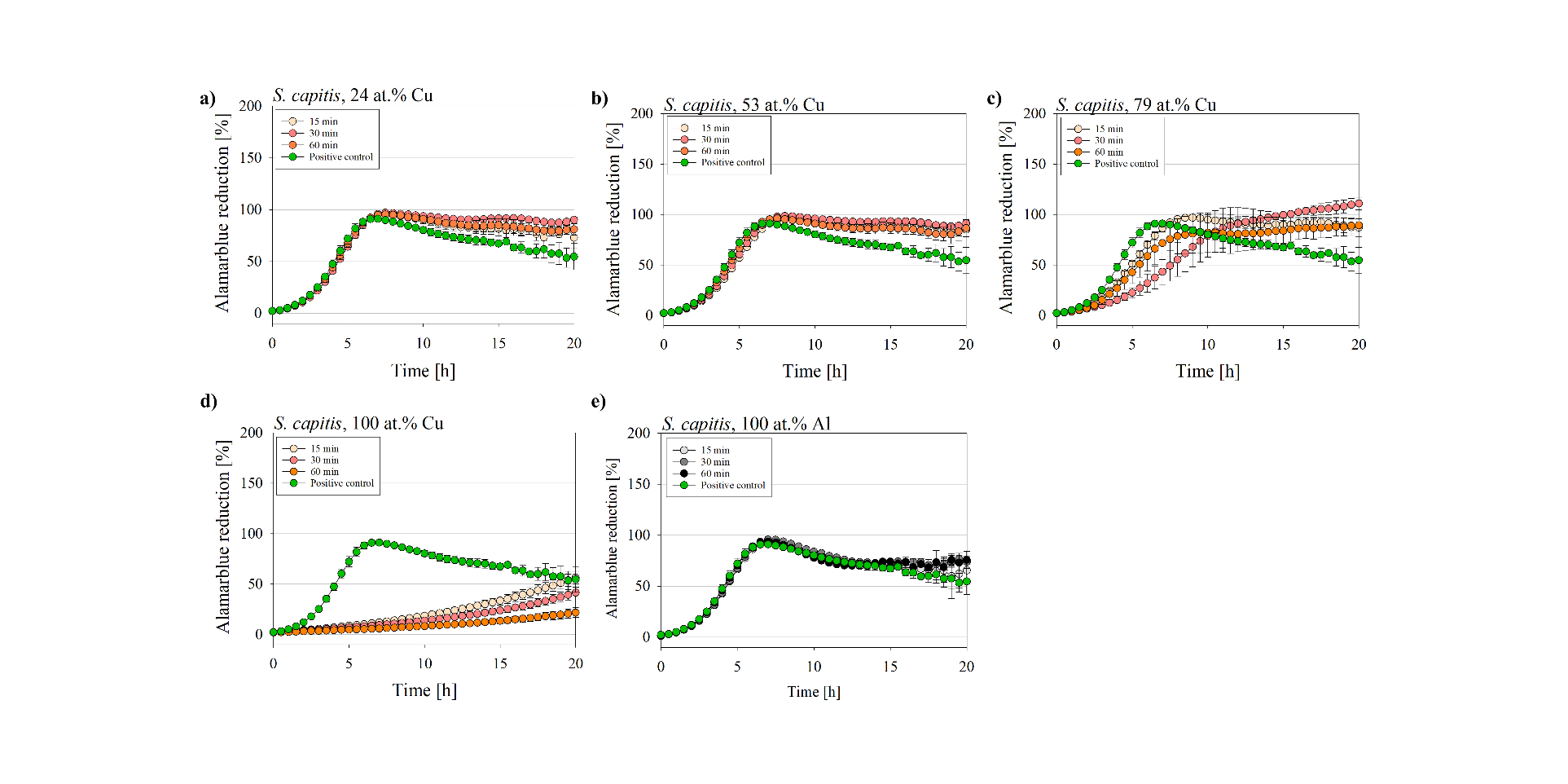
**Figure S1:** AlamarBlue assay with *S. capitis*. Metabolic activity of bacterial cells after contact with coatings with the following copper contents **a)** 24 at.%, **b)** 53 at.%, **c)** 79 at.%, **d)** 100 at.%, and on **e)** aluminum coatings. The data is displayed as mean and standard error (n=3).

**Figure S2:** AlamarBlue assay with *B. lata.* Metabolic activity of bacterial cells after contact with coatings with the following copper contents **a)** 24 at.%, **b)** 53 at.%, **c)** 79 at.%, **d)** 100 at.%, and on **e)** aluminum coatings. The data is displayed as mean and standard error (n=3).

In **Figure S2**, the results of the alamarBlue assay of *B. lata* are displayed. In contrast to *S. capitis*, the cells of *B. lata* showed a reduction of metabolic activity only after exposure on 100 at.% copper coatings (**Figure S2a-d**). Similarly to *S. capitis*, the exposure on Aluminum coatings (**Figure S2e**) led to a lowered metabolic reduction of 150 % compared to all exposure times on 24 at.% copper coatings.

**
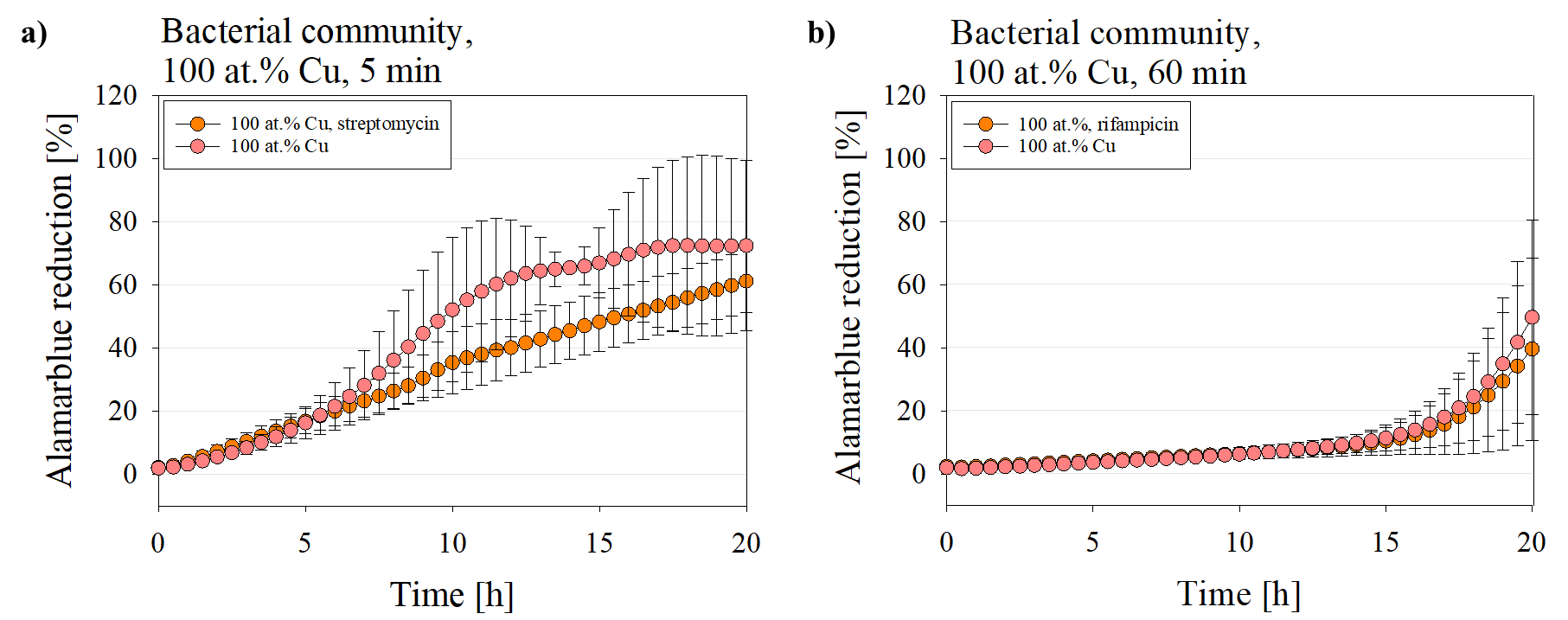
**

**Figure S3:** AlamarBlue assay with the bacterial community after wet contact killing on 100 at.% copper content surface coating. **a)** 5 min of wet contact killing, incubated in culture medium with streptomycin and **b)** 60 min of wet contact killing, incubated in culture medium with rifampicin. The data is displayed as mean and standard error (n=3).


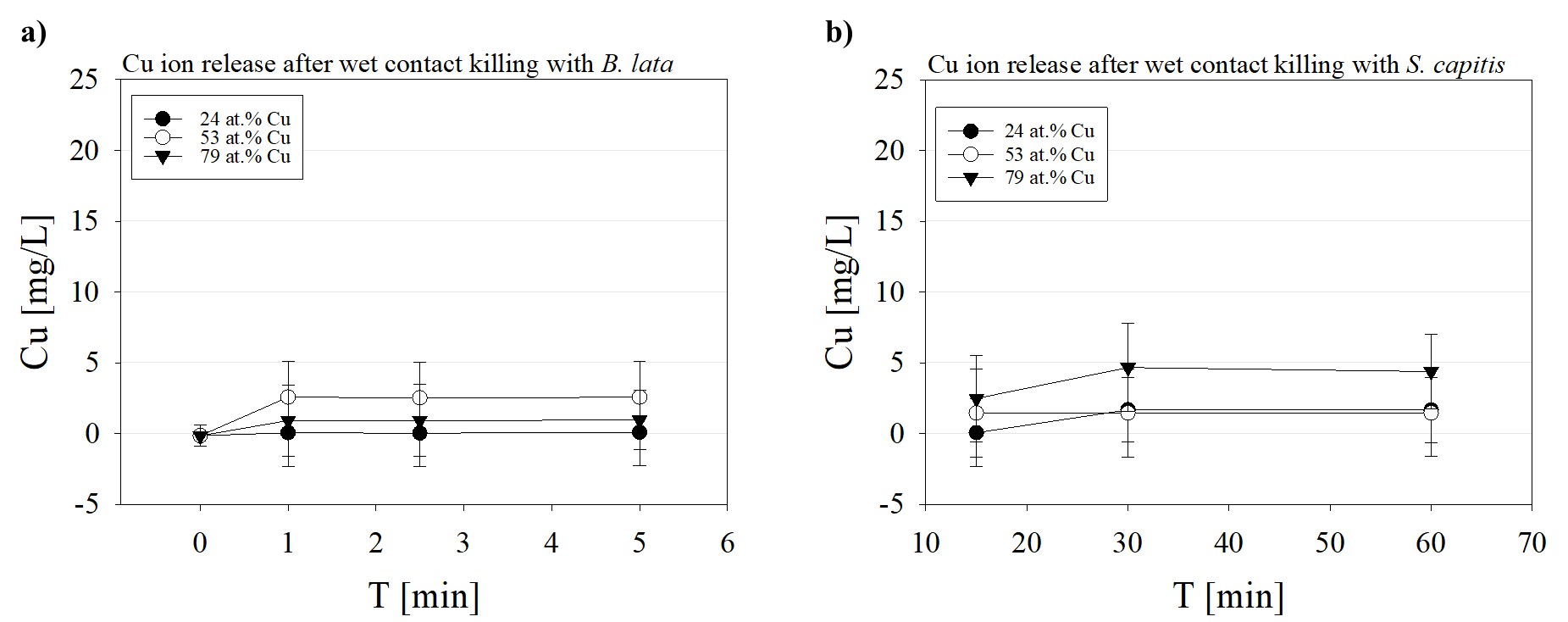
**Figure S4:** Copper ion release detection via ICP-MS after wet contact killing on surfaces with 24 at.% Cu, 53 at.% Cu, and 79 at.% Cu with **a)** *S. capitis* and **b)** *B. lata*.

| **Spectrum name** | **Spectrum 355** | **Spectrum 358** |
| --- | --- | --- |
| C | 34.02 | 42.80 |
| O | 34.25 | 30.02 |
| Na | 8.62 | 10.02 |
| Si | 2.51 | 1.50 |
| S | 3.74 | 4.84 |
| Cl | 1.39 |  |
| Cu | 8.91 | 5.27 |
| Zr | 6.56 | 5.55 |
| Total | 100.00 | 100.00 |


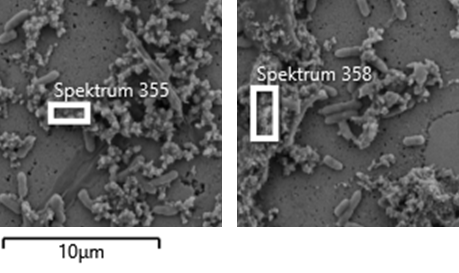


**Figure S5:** Composition of elements on extracellular material on 100 at.% Cu coatings after wet contact killing with *B. lata*.
